# Closing and opening of the RNA polymerase trigger loop

**DOI:** 10.1073/pnas.1920427117

**Published:** 2020-06-22

**Authors:** Abhishek Mazumder, Miaoxin Lin, Achillefs N. Kapanidis, Richard H. Ebright

**Affiliations:** ^a^Waksman Institute, Rutgers University, Piscataway, NJ 08854;; ^b^Department of Chemistry, Rutgers University, Piscataway, NJ 08854; ^c^Biological Physics Research Group, Clarendon Laboratory, Department of Physics, University of Oxford, OX1 3PU Oxford, United Kingdom

**Keywords:** RNA polymerase, trigger loop, transcription, conformational dynamics, single-molecule fluorescence resonance energy transfer

## Abstract

The RNA polymerase (RNAP) trigger loop (TL) is a mobile structural element of the RNAP active center that, based on crystal structures, has been proposed to cycle between an “unfolded”/“open” state that allows an NTP substrate to enter the active center and a “folded”/“closed” state that holds the NTP substrate in the active center. Here, by quantifying single-molecule fluorescence resonance energy transfer between a first fluorescent probe in the TL and a second fluorescent probe elsewhere in RNAP or in DNA, we detect and characterize TL closing and opening in solution. We show that the TL closes and opens on the millisecond timescale; we show that TL closing and opening provides a checkpoint for NTP complementarity, NTP ribo/deoxyribo identity, and NTP tri/di/monophosphate identity, and serves as a target for inhibitors; and we show that one cycle of TL closing and opening typically occurs in each nucleotide addition cycle in transcription elongation.

## Significance

During transcription elongation at saturating nucleotide concentrations, RNA polymerase (RNAP) performs ∼50 nucleotide-addition cycles every second. The RNAP active center contains a structural element, termed the trigger loop (TL), that has been suggested, but not previously shown, to open to allow a nucleotide to enter and then to close to hold the nucleotide in each nucleotide-addition cycle. Here, using single-molecule fluorescence spectroscopy to monitor distances between a probe incorporated into the TL and a probe incorporated elsewhere in the transcription elongation complex, we show that TL closing and opening occur in solution, define time scales and functional roles of TL closing and opening, and, most crucially, demonstrate that one cycle of TL closing and opening occurs in each nucleotide-addition cycle.

Transcription, the first and most highly regulated process in gene expression, entails transcription initiation, in which RNA polymerase (RNAP) binds to DNA and begins synthesis of an RNA molecule; followed by transcription elongation, in which RNAP extends the RNA molecule; followed by transcription termination, in which RNAP releases the RNA molecule (1–3). During transcription elongation, RNAP uses a “stepping” mechanism, in which RNAP translocates relative to DNA by one base pair for each nucleotide added to the RNA molecule (2, 4–6). Each “step” occurs as part of a “nucleotide-addition cycle” comprising: 1) RNAP translocation, 2) nucleoside-triphosphate (NTP) binding, 3) phosphodiester-bond formation, and 4) pyrophosphate release (2, 5, 7).

**Fig. 1.**
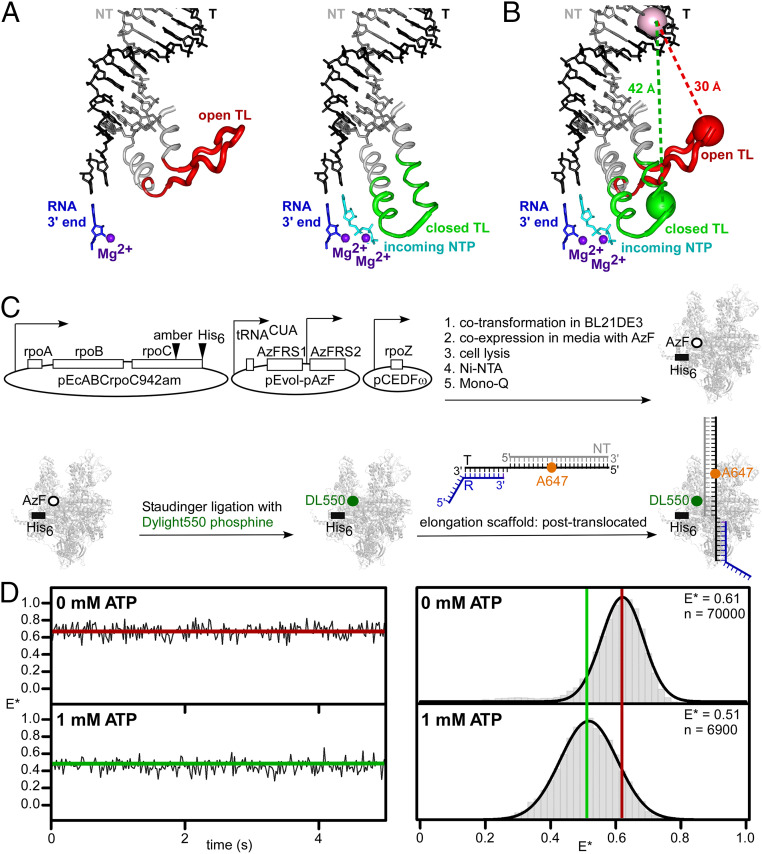
Use of smFRET to detect and characterize TL closing and opening in solution. (*A*) Open-TL (*Left*) and closed-TL (*Right*) conformational states as observed in crystal structures of *Thermus thermophilus* RNAP (refs. 9 and 29; Protein Data Bank [PDB] ID codes 1ZYR and 2O5J). Gray and red ribbon, RNAP trigger helices and TL in open-TL state; gray and green ribbon, RNAP trigger helices and TL in closed-TL state; gray and black sticks, DNA nontemplate and template strands; blue sticks, RNA 3′ nucleotide; cyan stick, incoming NTP; purple spheres, catalytic Mg^2+^ ions Mg^2+^(I) (*Left* and *Right*) and Mg^2+^(II) (*Right*). (*B*) Measurement of smFRET between first fluorescent probe incorporated at β′ residue 942 in tip of RNAP TL (red sphere for open-TL state; green sphere for closed-TL) and second fluorescent probe incorporated at template-strand position +12 of downstream DNA (pink sphere). Interresidue distances are ∼30 Å for open-TL state and ∼42 Å for closed-TL state. (*C*) Use of unnatural-amino acid mutagenesis (first reaction arrow), Staudinger ligation (second reaction arrow), and TEC reconstitution (third reaction arrow) to prepare sample for measurement of smFRET between first fluorescent probe incorporated at tip of RNAP TL and second fluorescent probe incorporated into downstream DNA (*Methods*). White open ovals, open rectangles, two-segment arrows, arrowhead labeled “amber,” and arrowhead labeled “His_6_” denote plasmids, genes, promoters, amber codon in TL coding sequence in gene for RNAP β′ subunit, and hexahistidine coding sequence at 3′ end of gene for RNAP β′ subunit. Gray ribbon structure, white open circle, green filled circle, and black bar denote RNAP, unnatural amino acid 4-azidophenylalanine in RNAP TL, fluorescent probe Dylight 550 in RNAP TL, and hexahistidine tag at C terminus of RNAP β′ subunit. Gray lines, black lines, blue lines, and orange filled circle denote nucleic-acid scaffold comprising nontemplate-strand DNA, template-strand DNA, RNA, and fluorescent probe Alexa647. (*D*) smFRET data for TEC in posttranslocated state in absence of NTP (*Upper*) and in presence of saturating concentration of complementary NTP (1 mM ATP; *Lower*). *Left*, representative time traces of donor-acceptor FRET efficiency, E*, showing open-TL (*Upper*, red) and closed-TL (*Lower*, green) states. *Right*, histograms and Gaussian fits of E*, showing mean E* values for open-TL (red line) and closed-TL (green line) states; n, number of frames.

A proposed key player in the nucleotide-addition cycle is the RNAP “trigger loop” (TL), a mobile structural element of the RNAP active center that is conserved in RNAP from bacteria through humans (2, 5, 8–11). Crystal structures of transcription elongation complexes indicate that the TL can adopt 1) an “unfolded,” or “open,” TL conformation that allows an NTP to enter the RNAP active center (observed in crystal structures without a bound NTP; refs. 2, 5, and 8–11, Fig. 1*A*, and *SI Appendix*, Fig. S1*A*); and 2) a “folded,” or “closed,” TL conformation that holds an NTP in the RNAP active center (observed in crystal structures with a bound NTP; refs. 2, 5, and 8–11, Fig. 1*A*, and *SI Appendix*, Fig. S1*A*). The open and closed TL conformations observed in crystal structures differ by a large—up to ∼20 Å—displacement of residues at the tip of the TL (refs. 2, 5, and 8–11, Fig. 1*B*, and *SI Appendix*, Fig. S1*B*). It has been hypothesized that the open and closed TL conformations observed in crystal structures occur in solution and are crucial for RNAP function in solution. Specifically, TL closing has been hypothesized to contribute to discrimination between complementary and noncomplementary NTPs and to contribute to discrimination between NTPs and dNTPs (2, 5, 8–28). TL closing also has been hypothesized to contribute to catalysis of phosphodiester-bond formation, by increasing the order of the RNAP active center, by excluding solvent from the RNAP active center, and by positioning residues to participate in electrostatic, general-acid/general-base, or conformational stabilization of the γ-phosphate/β-phosphate leaving group (2, 5, 8–28). It further has been hypothesized that the TL returns to its initial, unfolded, open conformational state on or after phosphodiester-bond formation, thereby reopening the RNAP active center and permitting pyrophosphate release and RNAP translocation (2, 5, 8–28).

According to these proposals, an RNAP active-center conformational cycle, comprising TL closing followed by TL opening, is coupled to each nucleotide-addition cycle (2, 5, 8–28). Consistent with these proposals, amino acid substitutions predicted to interfere with TL closing and opening (e.g., substitution of TL Gly residues with conformationally more restricted Ala residues) interfere with nucleotide addition (14–16, 18, 19, 25–27). Further consistent with these proposals, small molecules predicted to interact with, and trap, the TL in one conformational state interfere with nucleoti-de addition (9, 13, 14, 22, 29–36). Further consistent with these proposals, a “Cys-pair reporter strategy” detects differences in rates of disulfide-bond formation that correlate with predicted TL closed and open states (22, 25). However, no direct observation of TL closed and open states in solution has been reported. In particular, no direct observation of TL closing and opening during real-time, active transcription elongation in solution has been reported, and, as a result, it has not been possible to test directly the hypothesis that an RNAP active-center conformational cycle comprising TL closing followed by TL opening is coupled to each nucleotide-addition cycle.

Here, by use of single-molecule fluorescence resonance energy transfer (smFRET), we detect and characterize TL closing and opening in solution, including TL closing and opening during real-time, active transcription elongation in solution.

## Use of smFRET to Detect and Characterize TL Closing and Opening in Solution

In a first set of experiments, we assessed smFRET between a first fluorescent probe, serving as donor, in the RNAP TL and a second fluorescent probe, serving as acceptor, in DNA (Fig. 1*B* and *SI Appendix*, Fig. S1*B*). We used a procedure comprising: 1) incorporation of the fluorescent probe DyLight 550, serving as donor, in the RNAP TL, by use of unnatural-amino acid mutagenesis and Staudinger ligation (37–40); 2) incorporation of the fluorescent probe Alexa647, serving as acceptor, in the template strand of a nucleic-acid scaffold comprising a template-strand DNA oligonucleotide, a nontemplate-strand DNA oligonucleotide, and a nonextendable, 3′-deoxyribonucleotide-containing RNA oligonucleotide; 3) assembly and analysis of a doubly labeled transcription elongation complex (TEC) from the resulting labeled RNAP and labeled nucleic-acid scaffold; 4) immobilization of the doubly labeled TEC, through a hexahistidine tag on RNAP on a surface functionalized with anti-hexahistidine tag antibody; and 5) measurement of smFRET (Fig. 1*C* and *SI Appendix*, Figs. S2 and S3).

We evaluated 10 potential labeling sites in the TL of *Escherichia coli* RNAP: i.e., β′ subunit residues 933–942. Seven sites were located in a TL segment that is unfolded in the open-TL state but folded in the closed-TL state (β′ residues 933–939), and three sites were located in a TL segment—the TL tip—that is unfolded in both the open-TL and closed-TL states (β′ residues 940–942). All 10 sites were located in the TL region immediately preceding the species-specific sequence insertion present in the TL of *E. coli* RNAP (“SI3”; also referred to as “β′ G non-conserved domain”; β′ residues 943–1130; ref. 41). All 10 sites were sites that exhibit large, ∼15–25 Å, differences in Cα positions in crystal structures of the open-TL and closed-TL states (Fig. 1*A* and *SI Appendix*, Fig. S1). For each of the 10 sites, we prepared labeled RNAP and then assessed labeling efficiency, labeling specificity, and transcriptional activity. For three sites—β′ residues 940, 941, and 942—the labeled RNAP derivative retained ≥50% of the transcriptional activity of unlabeled WT RNAP, and, for one site—β′ residue 942—the labeled RNAP derivative retained ≥70% of the transcriptional activity of unlabeled WT RNAP (*SI Appendix*, Fig. S3). We performed subsequent experiments using RNAP labeled at β′ residue 942.

We evaluated two labeling sites in DNA, both located in the double-stranded DNA segment downstream of the RNAP active center: i.e., template-strand position +12 and template-strand position +10 (Fig. 1*B*). The two labeling sites in DNA were selected to provide the largest and second-largest predicted differences in interresidue distance, and the corresponding largest and second-largest predicted differences in smFRET, for the open-TL state vs. the closed-TL state (high FRET in open-TL state; low FRET in closed-TL state; Fig. 1*B* and *SI Appendix*, Fig. S1*B*). The labeling site at position +12 was used in most experiments; the labeling site at position +10 was used in selected additional experiments.

We analyzed TECs in two translocational states: 1) the pretranslocated state (i.e., the state at the start of the nucleotide-addition cycle, with an RNA-DNA hybrid of 10 bp, and with an RNAP active-center addition site—“A site”—occupied by the RNA 3′ end; nucleic-acid scaffold sequences in *SI Appendix*, Fig. S2*B*), and 2) the posttranslocated state (i.e., the state in which RNAP has stepped forward by 1 bp, reducing the length of the RNA-DNA hybrid to 9 bp, and rendering the RNAP active-center A site unoccupied and available to bind the next NTP; nucleic-acid scaffold sequences in *SI Appendix*, Fig. S2*A*). For analysis of the posttranslocated state, we employed a nonextendable, 3′-deoxyribonucleotide-containing RNA in order to allow NTP binding but not NTP addition.

To monitor the distance between fluorescent probes in the resulting donor-acceptor–labeled TECs, we used total internal reflection fluorescence microscopy with alternating laser excitation (TIRF-ALEX) (37, 39, 40, 42–45) and quantified smFRET from single TECs immobilized, through a hexahistidine tag on RNAP, on anti-hexahistidine-tag-antibody-functionalized glass coverslips. TIRF-ALEX allows filtering of data to identify only single molecules that contain both a donor and an acceptor, eliminating complications due to incomplete labeling and imperfect reconstitution of TECs (37, 39, 40, 42–45). The results provide equilibrium population distributions of apparent smFRET efficiency, E*. Monitoring E* time trajectories of individual TECs reports on the kinetics of TL conformational cycling.

For TECs in the posttranslocated state in the absence of an NTP, we observed unimodal E* distributions with mean E* of 0.61, corresponding to a probe-probe mean distance, R, of 49 Å (Fig. 1 *D*, *Upper Right*). There was no indication of any other FRET state within the temporal resolution of the experiment (∼20 ms), and there likewise was no indication of other FRET states in experiments employing confocal optical microscopy to provide higher temporal resolution (∼1 ms; *SI Appendix*, Fig. S4, *Right*). We conclude that, in a posttranslocated TEC state, in the absence of an NTP, the TL is predominantly, potentially exclusively, in a high-FRET state with mean E* of 0.61.

In contrast, for TECs in the posttranslocated state in the presence of a saturating concentration of the complementary NTP (ATP, using a template directing binding of ATP), the distribution was unimodal, but the mean E* was shifted from ∼0.61 to ∼0.51, indicative of a low-FRET state with a probe-probe mean distance, R, of ∼53 Å (Fig. 1 *D*, *Lower Right*). There was no indication of a high-FRET state within the temporal resolution of the experiment (∼20 ms) in these experiments. Qualitatively identical results were obtained in experiments using each of two labeling sites in DNA (template-strand positions +10 and +12; Fig. 1*D* and *SI Appendix*, Fig. S4). We conclude that, in a posttranslocated TEC, in the presence of a saturating concentration of the complementary NTP, the TL is predominantly, potentially exclusively, in a low-FRET state with mean E* of 0.51. Based on our expectation of a higher FRET value for the open-TL state than for the closed-TL state (Fig. 1*B* and *SI Appendix*, Fig. S1*B*), we assign the unimodal E* distribution for the posttranslocated TEC in the absence of NTP (mean E* ∼ 0.61; R ∼ 49 Å) to the open-TL state (Fig. 1 *D*, *Upper Right*), and we assign the unimodal E* distribution for the posttranslocated TEC in the presence of saturating complementary NTP, (mean E* ∼ 0.51; R ∼ 53 Å) to the closed-TL state (Fig. 1 *D*, *Lower Right*). We conclude further that our smFRET assay enables detection and differentiation of open-TL and closed-TL states in solution.

For TECs in the pretranslocated state, we observed unimodal E* distributions with mean E* of 0.62, indicative of an open-TL state (*SI Appendix*, Fig. S5 and Table S1), exactly as for TECs in the posttranslocated state in the absence of an NTP (Fig. 1 *D*, *Upper Right*). There was no indication of a lower-FRET, closed-TL state within our temporal resolution (∼20 ms). Assuming that the nucleic-acid scaffolds used here yield exclusively a pretranslocated state, we conclude that, in a pretranslocated TEC state, the TL is predominantly, potentially exclusively, in the open-TL state.

## TL Closing and Opening Occur on Millisecond Timescales

**Fig. 2.**
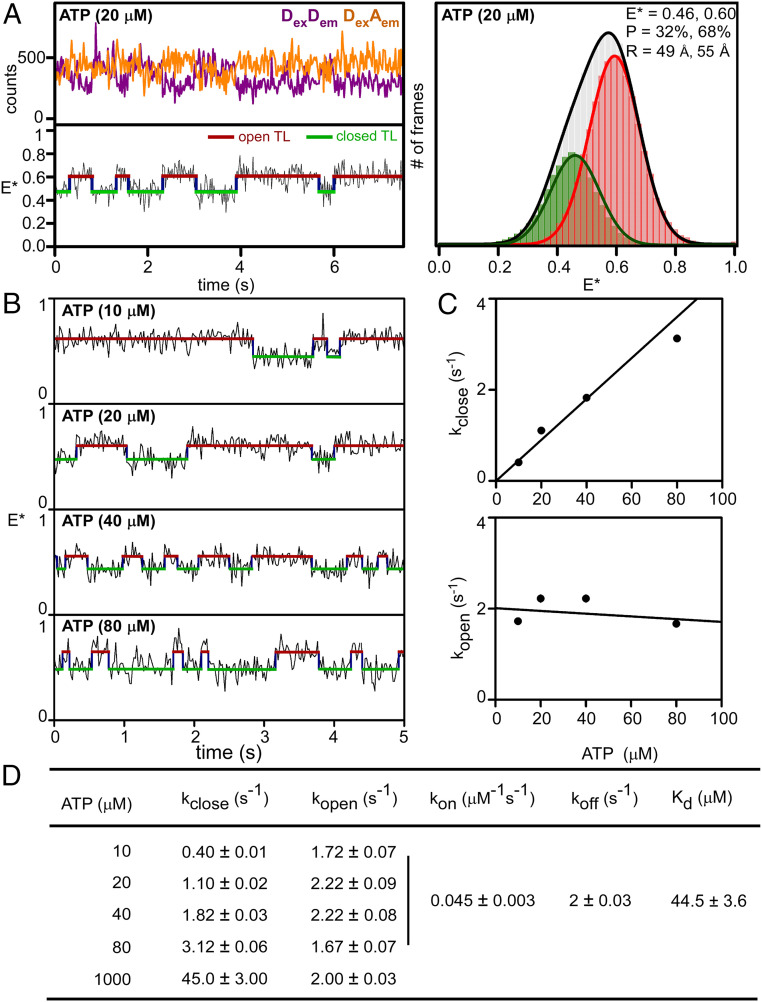
TL closing and opening occur on millisecond timescales. (*A*) smFRET data for TEC in posttranslocated state in presence of subsaturating concentration of complementary NTP (20 μM ATP). *Upper Left*, representative time trace of donor emission (purple) and acceptor emission (orange). *Lower Left*, representative time trace of donor-acceptor FRET efficiency, E*, showing HMM-assigned open-TL states (red), closed-TL states (green), and interstate transitions (blue). *Right*, histograms and Gaussian fits of E*. P, subpopulation percentage; R, mean donor-acceptor distance. (*B*) smFRET data for TEC in posttranslocated state in presence of each of four subsaturating concentrations of complementary NTP (10, 20, 40, and 80 μM ATP). Colors as in *A*, *Lower Left*. (*C*) ATP-concentration dependences of TL-closing rate (*k*_close_; *Upper*) and TL-opening rate (*k*_open_; *Lower*). (*D*) TL-closing rate (*k*_close_), TL-opening rate (*k*_open_), ATP on-rate (*k*_on_), ATP off-rate (*k*_off_), and ATP equilibrium dissociation constant (*K*_d_) from experiments of *A*–*C*.

In a next set of experiments, we examined TL conformation in the presence of subsaturating concentrations of the complementary NTP (ATP, using a template directing binding of ATP; Fig. 2 and *SI Appendix*, Fig. S6). Upon addition of a subsaturating concentration (20 μM) of the complementary NTP to posttranslocated TECs, E* time trajectories for a subpopulation of molecules (∼25%) showed transitions between two FRET states, indicating cycling between two TL conformational states (Fig. 2*A*). Hidden Markov Modeling (HMM) analysis of individual E* time trajectories identified two FRET states, one with E* of 0.60 (68%), corresponding to the open-TL state, and the other with E* of 0.46 (32%), corresponding to the closed-TL state (Fig. 2 *A*, *Right* and *SI Appendix*, Table S1). From single-exponential fits of dwell-time distributions for the two FRET states, we estimated lifetimes of the TL-open state (900 ms) and TL-closed state (500 ms), and estimated rates of TL opening (*k*_open_) and TL closing (*k*_close_) (Fig. 2 *B*–*D* and *SI Appendix*, Fig. S6 *B* and *C*). We next performed analogous experiments and analogous dwell-time analyses over a range of subsaturating concentrations of complementary NTP (10 μM, 20 μM, 40 μM, and 80 μM complementary NTP; Fig. 2 *B*–*D* and *SI Appendix*, Fig. S6). The results revealed that, with increasing concentrations of complementary NTP, *k*_close_ increases and *k*_open_ remains constant (Fig. 2 *C* and *D* and *SI Appendix*, Fig. S6). We conclude that binding of a complementary NTP induces TL closure, that TL-closing events correspond to NTP-binding events, and that TL-opening events correspond to NTP unbinding events. From the ATP concentration dependence of *k*_close_, we estimate the on-rate for ATP, *k*_on,ATP_, as 0.045 μM^−1^s^−1^; from the mean value of *k*_open_ and the assumption that TL opening is fast relative to ATP dissociation, we estimate the off-rate for ATP, *k*_off,ATP_, as 2 s^−1^; and, from the association and dissociation rate for ATP, we estimate the equilibrium dissociation constant for ATP, *K*_d,ATP_, as ∼45 μM (Fig. 2*D*). The estimated value of *K*_d,ATP_ (∼45 μM) is in excellent agreement with the published value of *K*_d,ATP_ (44.6 μM) (46).

## TL Closing and Opening Can Provide a Checkpoint for NTP Complementarity, Provide a Checkpoint for NTP Identity, and Serve as a Target for Inhibitors

**Fig. 3.**
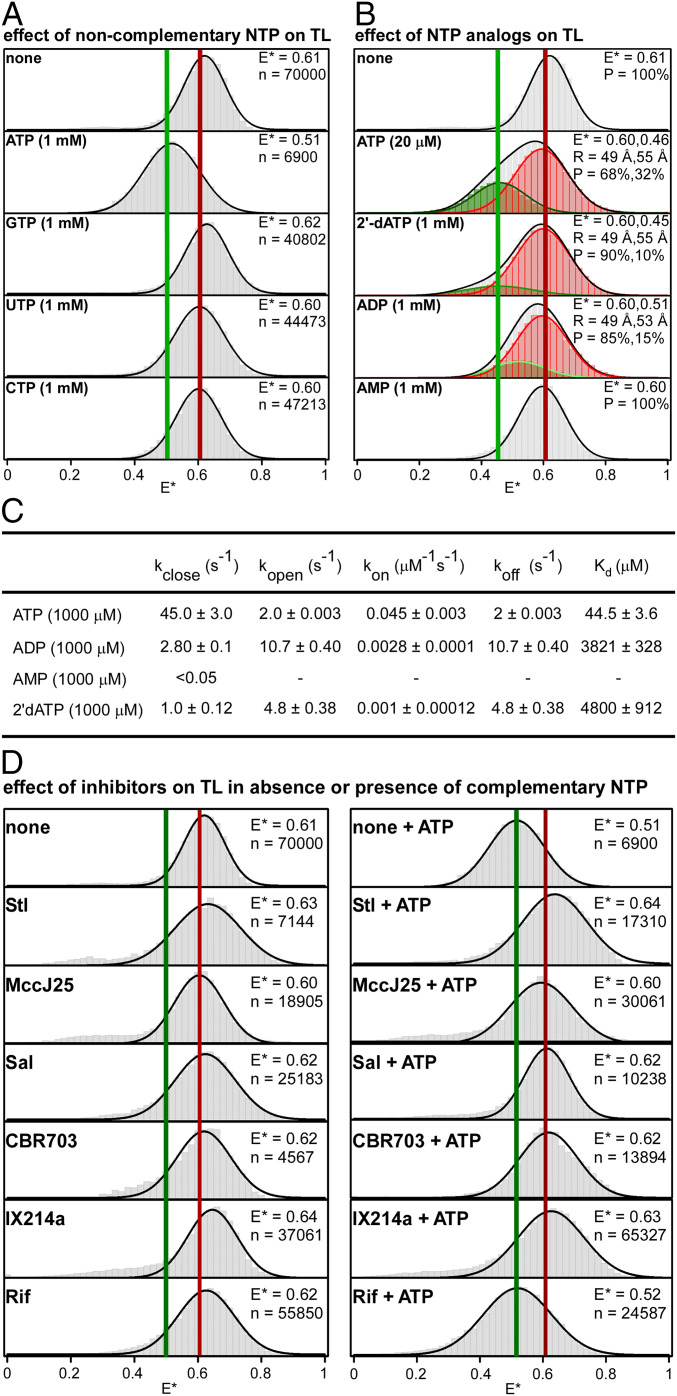
TL closing and opening can provide a checkpoint for NTP complementarity, provide a checkpoint for NTP identity, and serve as a target for inhibitors. (*A*) Effects of noncomplementary NTPs (GTP, UTP, and CTP for template directing incorporation of *A*) on TL conformation. Colors as in Fig. 1*D*, *Right*. (*B*) Effects of NTP analogs (dATP, ADP, and AMP) on TL conformation. Colors as in Fig. 2*A*, *Right*. (*C*) TL-closing rate (*k*_close_), TL-opening rate (*k*_open_), nucleotide association rate (*k*_on_), nucleotide dissociation rate (*k*_off_), and nucleotide equilibrium dissociation constant *K*_d_ from experiments of *B*. (*D*) Effects of small-molecule inhibitors of RNAP on TL conformation in absence of NTP (*Left*) and in presence of saturating concentration of complementary NTP (1 mM ATP; *Right*) CBR 703, CBR hydroxamidine CBR703; IX214a, *N*α-aroyl-*N*-aryl-phenylalaninamide IX-214a; MccJ25, microcin J25; Rif, rifampin. Colors as in Fig. 1*D*, *Right*.

In a next set of experiments, we assessed effects of noncomplementary NTPs on TL conformation (GTP, UTP, and CTP in experiments using a template directing binding of ATP; Fig. 3*A* and *SI Appendix*, Table S1). In the presence of 1 mM noncomplementary NTP, we observed unimodal E* distributions with E* of ∼0.60, corresponding to the open-TL state (Fig. 3*A* and *SI Appendix*, Table S1). There was no indication of a lower-FRET, closed-TL state within the temporal resolution of the experiment (∼20 ms) in presence of any noncomplementary NTP (Fig. 3*A*). Analogous results were obtained in analogous experiments using templates directing binding of GTP (ATP, UTP, and CTP as noncomplementary NTPs), directing binding of UTP (ATP, GTP, and CTP as noncomplementary NTPs), and directing binding of CTP (ATP, GTP, and UTP as noncomplementary NTPs) (*SI Appendix*, Fig. S7). We conclude that TL closing strongly discriminates between complementary and noncomplementary NTPs and, thus, that TL closing can serve as a checkpoint for NTP complementarity.

We next assessed effects of NTP ribo/deoxyribo identity on TL conformation (ATP vs. dATP, using a template directing binding of ATP; Fig. 3 *B* and *C*). The addition of dATP to 1 mM elicited only a very small change in the E* distribution, indicating that dATP is much less effective than ATP in inducing TL closure (Fig. 3*B*). Dwell-time analyses analogous to those performed with ATP (see previous section) indicated that the equilibrium dissociation constant for dATP is ∼100 times higher than the equilibrium dissociation constant for ATP (∼5,000 μM vs. ∼45 μM; Figs. 1*D* and 3*C*). We conclude that dATP induces TL closure only ∼1/100 as potently as ATP and, thus, that TL closure can serve as a checkpoint for NTP ribo/deoxyribo identity.

We next assessed effects of NTP tri/di/monophosphate identity on TL conformation (ATP vs. ADP vs. AMP, using a template directing binding of ATP; Fig. 3 *B* and *C*). The addition of ADP to 1 mM elicited only a very small change in the E* distribution, and the addition of AMP to 1 mM elicited no change in the E* distribution, indicating that ADP is much less effective than ATP in inducing TL closure and that AMP is ineffective in inducing TL closure (Fig. 3*B*). HMM analysis of individual E* time trajectories for experiments with 1 mM ADP identified two FRET states: one with E* of 0.60 (85%), corresponding to the open-TL state, and the other with E* of 0.51 (15%), corresponding to a partially closed-TL state. The observation of a partially closed-TL state in the presence of ADP suggests that ADP may bind to the RNAP active center and induce or stabilize an intermediate TL conformation with probe-probe distance of ∼53 Å (Fig. 3*B* and *SI Appendix*, Table S1). Dwell-time analyses for the open-TL and partially closed-TL states analogous to those performed with ATP (see previous section) indicated that the equilibrium dissociation constant for ADP is ∼100 times higher than the equilibrium dissociation constant for ATP (∼4,000 μM vs. ∼45 μM; Figs. 2*D* and 3*C*). We conclude that ADP induces TL closure only ∼1/100 as potently as ATP, that AMP does not induce TL closure, and, thus, that TL closure can serve as a checkpoint for NTP tri/di/monophosphate identity.

We next evaluated effects of five small-molecule RNAP inhibitors that, based on crystal structures, interact with sites on RNAP that include or overlap the TL (Fig. 3*D*). Streptolydigin (Stl) interacts with the RNAP bridge helix C-terminal hinge and the TL, making direct contacts with the open-TL state that potentially stabilize the open-TL state (9, 29, 30). Microcin J25 (MccJ25) interacts with the RNAP bridge helix N- and C-terminal hinges and the TL, making direct contacts with the open-TL state that potentially stabilize the open-TL state (36). Salinamide A (Sal) interacts with the RNAP bridge-helix N-terminal hinge in a manner that potentially sterically precludes TL closure (31). The CBR hydrazide CBR703 and the *N*α-aroyl-*N*-aryl-phenylalaninamide IX214a interact with the RNAP bridge-helix N-terminal cap in a manner that potentially sterically precludes TL closure (32–35). We observed that none of the five small-molecule RNAP inhibitors significantly affected E* distributions in the absence of the complementary NTP (ATP in these experiments, using a template directing binding of A: Fig. 3 *D*, *Left*), but that all five affected E* distributions in the presence of the complementary NTP, completely inhibiting TL closure in the presence of the complementary NTP (Fig. 3 *D*, *Right*). The inhibition of TL closure in the presence of the complementary NTP was observed only for inhibitors that interact with sites on RNAP that include or overlap the TL; no such inhibition was observed for an inhibitor, rifampin (Rif), that interacts with a site on RNAP that does not include or overlap the TL (ref. 47 and Fig. 3*D*). We conclude that the small-molecule inhibitors Stl, MccJ25, Sal, CBR703, and IX214a all inhibit TL closure in solution, and we conclude that the TL is a functional target for at least five classes of inhibitors in solution. We note that the smFRET assay of this report potentially could be adapted for high-throughput screening of TL-targeting small-molecule RNAP inhibitors.

## One TL Closing-Opening Cycle Typically Occurs for Each Nucleotide Addition in Transcription Elongation

**Fig. 4.**
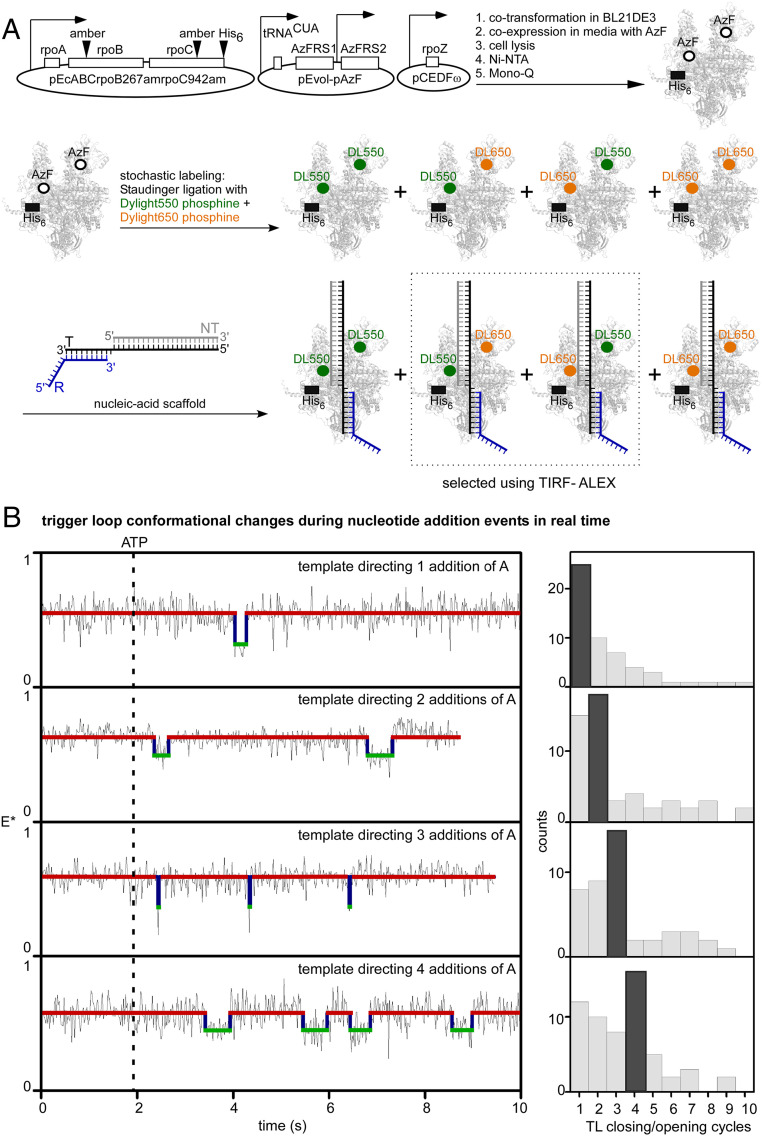
One TL closing-opening cycle typically occurs in each nucleotide addition in transcription elongation. (*A*) Use of unnatural-amino acid mutagenesis (first reaction arrow), Staudinger ligation with Dylight 550-phosphine and Dylight 650-phosphine (second reaction arrow), TEC reconstitution (third reaction arrow), and TIRF-ALEX to measure smFRET between first fluorescent probe at tip of RNAP TL and second fluorescent probe at reference site elsewhere in RNAP (*Methods*). Green filled circles, fluorescent probe Dylight 550; orange filled circles, fluorescent probe Dylight 650. Other colors as in Fig. 1*C*. (*B*) smFRET data for TEC engaged in active real-time transcription elongation on templates directing addition of 1, 2, 3, or 4 additions of *A*. *Left*, representative time traces of donor-acceptor FRET, E*. Black dashed line, addition of ATP. Other colors as in Fig. 2*A*, *Lower Left*. Among observed events, 31% have dwell times of ∼20 ms, 46% have dwell times between 40 ms and 300 ms, and 23% have dwell times >300 ms. *Right*, histograms showing numbers of detected TL closing/opening cycles. Note the one-for-one correlation between the modal number of TL closing/opening events detected for a template (dark gray bar for each template) and the number of nucleotide additions directed by the template.

We next monitored TL conformational cycling during real-time, active transcription elongation. To monitor TL conformational cycling during real-time, active transcription elongation, we prepared and analyzed TECs having a first fluorescent probe incorporated at a site in the RNAP TL (β′ residue 942; same site as in preceding sections) and having a second fluorescent probe incorporated at a reference site in RNAP (β residue 267; reference site selected to result in a large difference in interresidue distance, and a corresponding large predicted difference in smFRET, for the open-TL state vs. the closed-TL state; Fig. 4*A* and *SI Appendix*, Fig. S9). We used a procedure comprising: 1) incorporation of two fluorescent probes, DyLight 550 and Dylight 650, one serving as donor and the other as acceptor, at RNAP β′ residue 942 and RNAP β residue 267, by use of unnatural amino acid mutagenesis and Staudinger ligation (37, 39, 40); 2) assembly of a doubly labeled TEC from the resulting doubly labeled RNAP and a nucleic-acid scaffold comprising a template-strand DNA oligonucleotide programming one, two, three, or four additions of A, a nontemplate-strand DNA oligonucleotide, and an RNA oligonucleotide; 3) immobilization of the doubly labeled TEC, through a hexahistidine tag on RNAP, on an anti-hexahistidine tag-antibody-functionalized surface; and 4) measurement of smFRET using TIRF-ALEX (Fig. 4*A* and *SI Appendix*, Fig. S8).

To validate the use of this class of constructs, we performed smFRET experiments analogous to those in Figs. 1 and 2, assessing a construct of this class that directed binding of ATP and that contained a nonextendable 3′-deoxyribonucleotide–containing RNA (*SI Appendix*, Figs. S2*C* and S9). In the absence of a complementary NTP, we observed a unimodal E* distribution with mean E* of 0.59, indicative of an open-TL state with a probe-probe mean distance, R, of 53 Å (*SI Appendix*, Fig. S9 *B*, *Top* and *SI Appendix*, Table S2), and, in the presence of a saturating concentration of the complementary NTP (1 mM ATP), we observed a unimodal E* distribution with mean E* of 0.48, indicative of a closed-TL state with a probe-probe mean distance, R, of 58 Å (*SI Appendix*, Fig. S9 *B*, *Bottom* and *SI Appendix*, Table S2). In the presence of subsaturating concentrations of the complementary NTP (10 μM, 20 μM, 40 μM, and 80 μM ATP), we observed transitions between two FRET states: one with E* of 0.59, corresponding to the open-TL state, and the other with E* of 0.48, corresponding to the closed-TL state (*SI Appendix*, Fig. S9*C* and Table S2). Dwell-time analyses, performed as in the preceding sections, yielded estimates of *k*_on,ATP_ (0.038 μM^−1^s^−1^), *k*_off,ATP_ (biexponential fit; 17 s^−1^ and 2.4 s^−1^), and *K*_d,ATP_ (biexponential fit; 450 μM and 63 μM; *SI Appendix*, Fig. S9 *C* and *D*). The results demonstrate that this class of constructs enables detection and differentiation of TL-open and TL-closed states in solution.

We next assessed active transcription elongation, assessing constructs of this class that directed one addition of A, initiating transcription elongation by addition of ATP to 5 μM, and monitoring smFRET for 15 s (Fig. 4*B* and *SI Appendix*, Figs. S2 *C* and S10; observation time limited to ∼15 s by probe photobleaching at longer times). Approximately 25% of the molecules showed an unambiguous TL closing/opening cycle (characterized by a decrease in E* from ∼0.6 to ∼0.5 followed by an increase in E* from ∼0.5 to ∼0.6, each showing anticorrelated changes in signals in donor and acceptor channels) within the 15-s observation time (Fig. 4*B*). We extracted dwell-time distributions for the two FRET states, estimated lifetimes of the TL-open state (1,030 ms) and the TL-closed state (20 ms, 97% and 238 ms, 3%; biexponential fit), and estimated rates of TL opening (*k*_open_) and TL closing (*k*_close_) (*SI Appendix*, Fig. S10*A*).

We note that the mean dwell time for the TL-closed state in experiments assessing active transcription (1/*k*_open_; 20 ms; *SI Appendix*, Fig. S10*A*) is only ∼1/3 the mean dwell time for the TL-closed state in experiments assessing NTP binding (1/*k*_open_; ∼55 ms; *SI Appendix*, Fig. S9*D*). We suggest that this difference relates to the fact that the mean dwell time for the TL-closed state in experiments assessing active transcription (Fig. 4) corresponds to reactions from TL closing through either phosphodiester-bond formation and TL opening, or NTP dissociation and TL opening, whichever comes first, whereas the mean dwell time for the TL-closed state in experiments assessing NTP binding (Fig. 2) corresponds to reactions from TL closing through NTP dissociation and TL opening. Assuming that the mean dwell time for the TL-closed state in experiments assessing active transcription at 5 μM ATP approximates the mean time for reactions from TL closing through phosphodiester-bond formation and TL opening (1/*k*_open_; 20 ms; *SI Appendix*, Fig. S10*A*), and, assuming that the mean dwell time for the TL-open state in experiments assessing active transcription at 5 μM ATP approximates the mean time for the other reactions of the nucleotide-addition cycle (1/*k*_close_; 1,030 ms; *SI Appendix*, Fig. S10*A*), we estimate that the mean total duration of a nucleotide-addition cycle at 5 μM ATP is 1,050 ms. This estimate is in excellent agreement with the reported value for the mean total duration of a nucleotide-addition cycle at 5 μM ATP: ∼1,000 ms (4).

We next counted the number of TL closing/opening events for constructs of this class that directed one, two, three, or four additions of A, initiating transcription elongation by the addition of ATP to 5 μM at t = 0, and monitoring smFRET for 15 s (Fig. 4*B* and *SI Appendix*, Figs. S2*C* and S10*A*). We observed a remarkable, one-for-one correlation between the modal number of TL closing/opening events detected for a nucleic-acid scaffold and the number of nucleotide additions directed by the nucleic-acid scaffold (Fig. 4*B*); the modal numbers of TL closing/opening events detected were one, two, three, and four for nucleic-acid scaffolds directing, respectively, one, two, three, and four nucleotide additions (Fig. 4 *B*, *Right*). We conclude that one cycle of TL closing/opening typically occurs for each nucleotide-addition step in transcription elongation.

Most, possibly all, cases in which the number of detected TL closing/opening events was lower than the modal number (∼30% of molecules) may represent cases where events occurred but were unresolved within our ∼20-ms temporal resolution; analysis of dwell-time distributions indicates that ∼60% of events are likely to have durations below 20 ms (*SI Appendix*, Fig. S10*A*). At least some cases in which the numbers of detected TL closing/opening events was higher than the modal number (∼30% of molecules) may represent cases where events occurred that did not result in net nucleotide addition, such as, for example, events where nucleotide addition was followed by nucleotide removal by pyrophosphorolysis or hydrolysis (2). Accordingly, we suggest that one cycle of TL closing/opening may always, or almost always, occur for each nucleotide addition in transcription elongation.

## Prospect

Our results demonstrate the direct detection of TL conformation and conformational cycling in solution (Figs. 1 and 2); show that the TL is open in the absence of an NTP in solution (Figs. 1*D* and 2); show that the TL closes upon binding an NTP in solution (Figs. 1*D* and 2); show that TL closing and opening occur on the millisecond time scale in solution (Fig. 2); show that the TL serves as a checkpoint for NTP complementarity, NTP ribo/deoxyribo identity, and tri/di/monophosphate identity (Fig. 3 *A*–*C*); show that the TL serves as a target for five classes of small-molecule RNAP inhibitors (Fig. 3*D*); and, most important, shows that, in most, and possibly all, cases, one TL closing/opening cycle occurs in each nucleotide-addition step during transcription elongation (Fig. 4). Our results provide direct support for the hypothesis from crystal structures (2, 5, 8–11) that the RNAP TL undergoes two large-amplitude conformational changes—first closing, with movement by ∼15–25 Å, and then opening, with movement by ∼15–25 Å—in each millisecond-timescale nucleotide-addition step in transcription elongation. Our results also provide direct support for the hypothesis from crystal structures (2, 5, 8–11) that RNAP TL conformational cycling is functionally important for substrate specificity and catalysis. Our results imply—in view of the TL's location near the center of the RNAP molecule, its conformational cycling, and its functional importance—that the TL is the veritable “beating heart” of RNAP.

Because the TL is conserved in RNAP from all living organisms, our conclusions regarding TL conformational cycling and TL functional importance are likely to be valid for RNAP from bacteria through humans, and our smFRET approach for analysis of TL conformation is likely to be applicable to RNAP from bacteria through humans.

We note that combining our smFRET approach with optical-tweezer or nanopore-tweezer approaches able to detect “stepping” of RNAP at single base-pair resolution (4, 48) could enable analysis of TL conformation and TL conformational cycling as a function of template location and template sequence in transcription elongation, transcriptional pausing, and transcription termination.

## Materials and Methods

Fluorescent probes were incorporated at specific sites in RNAP by use of unnatural-amino acid mutagenesis and Staudinger ligation (37–40). smFRET data were collected by use of TIRF-ALEX (37, 39, 40, and 42–45).

Full details of methods are presented in *SI Appendix*, *SI Materials and Methods*.

### Materials and Data Availability.

Data necessary for replication are included in the submission. MATLAB software packages Twotone-ALEX and ebFRET are available, respectively, at https://kapanidis.web.ox.ac.uk/software and on GitHub (http://ebfret.github.io/).

## Supplementary Material

Supplementary File
