# Supplementary material for: Closing and opening of the RNA polymerase trigger loop
Source: Proc Natl Acad Sci U S A. 2020 Jun 22;117(27):15642–9. doi: 10.1073/pnas.1920427117 (PMC7355006; doi:10.1073/pnas.1920427117)
Supplement: Supplementary File [file pnas.1920427117.sapp.pdf]

Materials and Methods:

RNAP derivatives

For experiments in Figs. 1-3 and S3-S7, fluorescent-probe-labelled, hexahistidine-tagged *Escherichia coli* RNAP core enzyme was prepared using unnatural-amino-acid mutagenesis (*1*) of co-expressed genes encoding RNAP β', β, α, and ω subunits to afford an RNAP core enzyme derivative containing 4-azido-L-phenylalanine (AzF) at position 942 of β', followed by azide-specific Staudinger ligation to incorporate the fluorescent probe Dylight 550 (DL550) at position 942 of β', as follows (Figs. 1C, S3; *2-4*): Single colonies of *E. coli* strain BL21(DE3) (Millipore) co-transformed with plasmid pEcABC-rpoC942am-His_6 [constructed from plasmid pEcABC-His_6 (*5*) by use of site-directed mutagenesis (QuikChange Site-Directed Mutagenesis Kit; Agilent) to replace *rpoC* codon 942 by an amber codon], plasmid pCDFω (*6*), and plasmid pEVOL-pAzF (*1*) were used to inoculate 20 ml LB broth (*7*) containing 100 μg/ml ampicillin, 50 μg/ml kanamycin, and 35 μg/ml chloramphenicol, and cultures were incubated 16 h at 37°C with shaking. Culture aliquots (2x10 ml) were used to inoculate LB broth (2x1 L) containing 2 mM AzF (Chem-Impex International), 100 μg/ml ampicillin, 50 μg/ml kanamycin, and 35 μg/ml chloramphenicol; cultures were incubated at 37°C with shaking until OD_{600} = 0.6; L-arabinose was added to 0.2% and IPTG was added to 1 mM; and cultures were further incubated 16 h at 16°C with shaking. Cells were harvested by centrifugation (4,000 x g; 20 min at 4˚C), re-suspended in 20 ml buffer A (10 mM Tris-HCl, pH 7.9, 200 mM NaCl, and 5% glycerol), and lysed using an EmulsiFlex-C5 cell disrupter (Avestin). The lysate was cleared by centrifugation (20,000 x g; 30 min at 4°C), precipitated with polyethyleneimine (Sigma-Aldrich) as in (*8*), and precipitated with ammonium sulfate as in (*8*). The precipitate was dissolved in 30 ml buffer A and loaded onto a 5 ml column of Ni-NTA-agarose (Qiagen) pre-equilibrated in buffer A, and the column was washed with 50 ml buffer A containing 10 mM imidazole and eluted with 25 ml buffer A containing 200 mM imidazole. The sample was further purified by anion-exchange chromatography on Mono Q 10/100 GL (GE Healthcare; 160 ml linear gradient of 300-500 mM NaCl in 10 mM Tris-HCl, pH 7.9, 0.1 mM EDTA, and 5% glycerol; flow rate = 2 ml/min). Fractions containing AzF-derivatized hexahistidine-tagged *E. coli* RNAP core enzyme were pooled, concentrated to ~1 mg/ml using 30 kDa MWCO Amicon Ultra-15 centrifugal ultrafilters (EMD Millipore), and stored in aliquots at -80°C. A reaction mixture containing 10 μM AzF-derivatized hexahistidine-tagged *E. coli* RNAP core enzyme and 250 μM DL550 phosphine (Thermo Fisher Scientific; Cat. no. 88910) in 1 ml buffer B (50 mM Tris-HCl, pH 7.9, 100 mM KCl, 5% glycerol, and 2% dimethylformamide) was incubated 1 h at 15°C, incubated 16 h on ice, and subjected to 5 cycles of buffer exchange (dilution with 5 ml buffer B, followed by concentration to 0.5 ml using 30 kDa MWCO Amicon Ultra-15 centrifugal ultrafilters). The sample was further purified by gel-filtration chromatography on HiLoad 16/60 Superdex 200 prep grade (GE Healthcare) pre-equilibrated in buffer C (20 mM Tris-HCl, pH 8.0, 100 mM NaCl, 5 mM $MgCl_2$, 1 mM β-mercaptoethanol, and 5% glycerol) and eluted in buffer C. Fractions containing fluorescent-probe-labelled, hexahistidine-tagged *E. coli* RNAP core enzyme were pooled, concentrated to 1 mg/ml in buffer C using 30 kDa MWCO Amicon Ultra-15 centrifugal ultrafilters, and stored in aliquots at -80°C.

For experiments in Figs. 4 and S8-S10, fluorescent-probe-labelled, hexahistidine-tagged *E. coli* RNAP core enzyme was prepared using unnatural-amino-acid mutagenesis (*1*) of co-expressed genes encoding RNAP β', β, α, and ω subunits to afford an RNAP core enzyme derivative containing AzF at position 942 of β' and position 267 of β, followed by stochastic azide-specific Staudinger ligation to incorporate the fluorescent probes Dylight 550 (DL550) and Dylight 650 (DL650) at position 942 of β' and position 267 of β (Figs. 4A, S8; *2-3*). The RNAP core enzyme derivative containing AzF at position 942 of β' and position 267 of β was prepared as described in the preceding paragraph for the RNAP core derivative containing AzF at position 942 of β', but using plasmid pEcABC-rpoB267am;rpoC942am-His_6 [constructed from plasmid pEcABC-rpoC942am-His_6 by use of site-directed mutagenesis (QuikChange Site-Directed Mutagenesis Kit; Agilent) to replace *rpoB* codon 267 by an amber codon] in place of plasmid pEcABC-rpoC942am-His_6. A reaction mixture containing 10 μM AzF-derivatized, hexahistidine-tagged *E. coli* RNAP core enzyme, 1000 μM

www.pnas.org/cgi/doi/10.1073/pnas.1920427117

Dylight 550 phosphine (Thermo Fisher Scientific; Cat. no. 88910), and 500 μM Dylight 650 phosphine (Thermo Fisher Scientific; Cat. no. 88911) in 1 ml buffer B (50 mM Tris-HCl, pH 7.9, 100 mM KCl, 5% glycerol, and 2% dimethylformamide) was incubated 1 h at 15°C, incubated 16 h on ice, subjected to 5 cycles of buffer exchange (dilution with 5 ml buffer B, followed by concentration to 0.5 ml) using 30 kDa MWCO Amicon Ultra-15 centrifugal ultrafilters (EMD Millipore), and stored in aliquots at -80°C.

Efficiencies of incorporation of fluorescent probes were determined from UV/Vis-absorbance measurements and were calculated as:

concentration of product = $[A_{280} - \varepsilon_{DL550,280} (A_{DL550,562}/\varepsilon_{Cy3B,562}) - \varepsilon_{DL650,280} (A_{DL650,660}/\varepsilon_{DL650,660})]/\varepsilon_{P,280}$
DL550 labelling efficiency = 100% $[(A_{DL550,562}/\varepsilon_{DL550,562})$/(concentration of product)]
DL650 labelling efficiency = 100% $[(A_{DL650,660}/\varepsilon_{DL650,660})$/(concentration of product)]

where A_{280} is the measured absorbance at 280 nm, $A_{DL550,562}$ is the measured absorbance at the long-wavelength absorbance maximum of DL550 (562 nm), $A_{DL650,660}$ is the measured absorbance at the long-wavelength absorbance maximum of DL650 (660 nm), $\varepsilon_{P,280}$ is the molar extinction coefficient of RNAP core enzyme at 280 nm (198,500 M^{-1} cm^{-1}), $\varepsilon_{DL550,280}$ is the molar extinction coefficient of DL550 at 280 nm (12,090 M^{-1} cm^{-1}), $\varepsilon_{DL650,280}$ is the molar extinction coefficient of DL 650 at 280 nm (9,250 M^{-1} cm^{-1}), $\varepsilon_{DL550,562}$ is the extinction coefficient of DL550 at its long-wavelength absorbance maximum (150,000 M^{-1} cm^{-1}), and $\varepsilon_{DL650,660}$ is the extinction coefficient of DL650 at its long-wavelength absorbance maximum (250,000 M^{-1} cm^{-1}). Labelling efficiencies were ~60% for DL550 for the singly labelled RNAP derivative (Figs. S3) and ~50% for DL550 and ~80% for DL650 for the doubly labelled RNAP derivative (Fig. S8).

Specificities of incorporation of fluorescent probes were determined from the observed labelling efficiencies of (i) the labelling reaction with the AzF-derivatized, hexahistidine-tagged *E. coli* RNAP core enzyme and (ii) a control labelling reaction with non-AzF-derivatized, hexahistidine-tagged *E. coli* RNAP core enzyme (prepared as described in *9*), and were calculated as:

labelling specificity =100% [1- [(labelling efficiency with P)/ (labelling efficiency with AzF-P)]

where AzF-P is AzF-derivatized, hexahistidine-tagged *E. coli* RNAP core enzyme, and P is non-AzF-derivatized, hexahistidine-tagged *E. coli* RNAP core enzyme. Labelling specificities were >90% (Figs. S3, S8).

Transcriptional activities of labelled RNAP derivatives were determined using fluorescence-detected transcription assays as described in (*10)* and are expressed relative to transcriptional activity of unlabelled *E. coli* RNAP measured under identical conditions.

σ^{70}
E. coli σ^{70} was prepared as in (*11*).

Nucleic acids
Oligodeoxyribonucleotides (Integrated DNA Technologies, Inc) and oligoribonucleotides (Trilink, Inc) were dissolved in nuclease-free water (Ambion, Inc) to a final concentration of 100 mM and stored at -20ºC. Oligodeoxyribonucleotides were labelled with Alexa Fluor 647 N-hydroxysuccinimide (NHS) ester (Molecular Probes) as described (*12*).

Nucleic-acid scaffolds
Nucleic-acid scaffolds (Fig. S2) were prepared as follows: Nontemplate-strand oligodeoxyribonucleotide (3 mM), template-strand oligodeoxyribonucleotide (2 mM), and oligoribonucleotide (8 mM) in 50 μl 10 mM Tris-HCl, pH 7.9 and 0.2 M NaCl were heated 5 min at

95°C, cooled to 25°C in 2°C steps with 1 min per step using a thermal cycler (Applied Biosystems), and stored at −20°C.

Small molecules

NTPs (Thermo Fisher Scientific, Cat. No. R0481), dATP (Thermo Fisher Scientific, Cat. No. R0141), ADP (Sigma-Aldrich), and AMP (Sigma-Aldrich) were diluted in nuclease-free water (Ambion, Inc) to final concentrations of 25 mM and stored in aliquots at -80ºC.

Streptolydigin (Stl) was the kind gift of Dr. E. Steinbrecher (Upjohn-Pharmacia, Kalamazoo, MI), and Salinamide A (Sal A) was the kind gift of Dr. W. Fenical (The Scripps Research Institute, La Jolla, CA). Rif was purchased from Sigma-Aldrich, and CBR703 was purchased from Maybridge. MccJ25 was prepared as in (*13*), and IX214A was prepared as in (*14*).

Transcription elongation complexes (TECs)

Fluorescent-probe-labelled, hexahistidine-tagged transcription elongation complexes (TECs) were prepared as follows: Reaction mixtures containing 10 nM single fluorescent-probe-labelled, hexahistidine-tagged *E. coli* RNAP core enzyme and 100 nM fluorescent-probe labelled nucleic-acid scaffold (for experiments in Figs. 1C, 2, 3 and Figs. S4-S7; sequences in Figs. S1A-B), or 10 nM fluorescent-probe-doubly-labelled, hexahistidine-tagged *E. coli* RNAP core enzyme and 100 nM nucleic-acid scaffold (for experiments in Figs. 4 and Figs. S9-S10; sequences in Figs. S2C) in 0.5 ml KG7 (40 mM HEPES-NaOH, pH 7.0, 100 mM potassium glutamate, 10 mM $MgCl_2$, 1 mM dithiothreitol, 100 μg/ml bovine serum albumin, and 5% glycerol) were incubated 15 min at 22ºC. Reaction mixtures then were concentrated to 0.05 ml and subjected to by 3 cycles of buffer exchange (dilution with 0.5 ml buffer KG7, followed by concentration to 0.05 ml) using 100 kDa MWCO Amicon Ultra-0.5 centrifugal filters (Millipore). TECs prepared using this procedure were stable for up to 24 h on ice.

smFRET using confocal-ALEX

Confocal-ALEX experiments were performed essentially as described (*2, 12*). A green laser (532 nm; Compass 215M-20; Coherent) was used for direct excitation of the donor, and a red laser (638 nm; Radius 635-25; Coherent,) was used for direct excitation of the acceptor (*2*). Lasers were operated at continuous-wave excitation intensities of 120 μW at 532 nm and 80 μW at 638 nm and were alternated at 25 μs intervals using an acousto-optical modulator (Neos Technologies, Inc.). Fiber-coupled collimated beams were directed to an Olympus IX71 inverted microscope (Olympus America, Inc.), reflected by a beam splitter, and focused into the sample through a 60x oil-immersion objective. Fluorescence emission from the sample was collected through the objective, filtered through a 100 μm pinhole, spectrally split by a dichroic mirror, and focussed onto two avalanche photodiode detectors (APD; SPCM-AQR-15; Perkin-Elmer).

For experiments in Figs. S4 and S7, TECs were diluted to a final concentration of 0.1 nM in 50 μl KG7-trolox [KG7 containing 2 mM Trolox (Sigma Aldrich)] containing the following nucleotides: (i) none; (ii) 1 mM ATP; (iii) 1 mM GTP; (iv) 1 mM CTP, or (v) 1 mM UTP. Following incubation 5 min at 22ºC, smFRET data collection was performed. Data-acquisition times ranged from 20-30 minutes.

Photons detected at the donor-emission channel upon donor excitation (F_{DD}), acceptor-emission channel upon donor excitation (F_{DA}), and acceptor-emission channel upon acceptor excitation (F_{AA}) were extracted based on photon arrival times. The stoichiometry parameter (S) was calculated for each above-threshold photon burst, as follows (*2, 12, 15*):

$$S = (F_{DA} + F_{DD})/ (F_{DD} + F_{DA} + F_{AA})$$

The donor-acceptor smFRET efficiency (E*) for each above-threshold, photon burst was calculated as follows (*2, 12, 15*):

$E^* = F_{DA} / (F_{DD} + F_{DA})$

Two-dimensional E*-S plots were used to distinguish species containing donor only (D-only), acceptor only (A-only), and both donor and acceptor (D-A). For species containing both donor and acceptor (D-A), one-dimensional E* histograms were plotted and were fitted with Gaussian curves (Figs. S4 and S7). The resulting histograms provided equilibrium population distributions of E*.

smFRET using TIRF-ALEX: sample preparation

Observation wells for smFRET experiments were prepared as described (*3-4*). Briefly, a biotin-PEG-passivated glass surface was prepared, functionalized with Neutravidin (Sigma Aldrich), and treated with biotinylated anti-hexahistidine monoclonal antibody (Penta-His Biotin Conjugate; Qiagen), yielding wells with (biotinylated anti-hexahistidine monoclonal antibody)-Neutravidin-biotin-PEG-functionalised glass floors.

For experiments in Figs. 1C, 2-4 and Figs. S4-S7 and S9-10, fluorescent-probe-labelled, hexahistidine tagged TECs were immobilised in observation wells with (biotinylated anti-hexahistidine monoclonal antibody)-Neutravidin-biotin-PEG-functionalized glass floors, as follows: aliquots (30 μl) of 0.1 nM fluorescent-probe-labelled, hexahistidine tagged TEC in KG7 were added to the observation chamber and incubated 2-4 min at 22ºC, solutions were removed, wells were washed with 2X30 μl KG7, and 30 μl imaging buffer [KG7 containing 2 mM Trolox (Sigma-Aldrich), 12.5 μM glucose oxidase (Sigma-Aldrich), 16 nM catalase (from bovine liver C30; Sigma-Aldrich), and 8 mM D-glucose] at 22ºC was added. Immobilization densities typically were ~20-30 molecules per 10 μm x 12 μm field of view (assessed by manual counting). Immobilization specificities typically were >98% (assessed in control experiments omitting biotinylated anti-hexahistidine monoclonal antibody).

For experiments in Fig. 3D in the absence of NTPs, observation chambers containing immobilized fluorescent-probe-labelled, hexahistidine-tagged TECs were prepared as described above, except that (i) 1 nM fluorescent-probe-labelled, hexahistidine-tagged TEC was pre-incubated 5 min at 22°C in KG7 containing the specified concentration of inhibitor (none, 20 μM Rif, 50 μM Stl, 100 μM MccJ25, 20 μM Sal, 50 μM CBR703, or 200 μM IX214A) and then diluted 1:10 with KG7 containing the specified concentration of inhibitor before addition of aliquots to observation chambers, and (ii) the KG7 wash stocks contained the specified concentration of inhibitor, and (iii) the imaging buffer stocks contained the specified concentration of inhibitors.

For experiments in Figs. 1C, 2, 3A-C, S6, and S9 in the presence of NTPs or NTP analogs, observation chambers containing immobilized fluorescent-probe-labelled, hexahistidine-tagged TECs (prepared as described above) were supplemented to the specified final concentrations with 30 μl solutions of NTPs or NTP analogs in imaging buffer, reaction mixtures were incubated 3 min at 22°C, and data were collected.

For experiments in Figs. 4 and S10, observation chambers containing immobilized fluorescent-probe-labelled, hexahistidine-tagged TECs were prepared as described above with 30 μl imaging buffer in the observation chambers, data acquisition was started, and observation chambers were supplemented with 10μl of 20μM ATP in imaging buffer (yielding a final ATP concentration of 5 μM).

smFRET using TIRF-ALEX: data collection and data analysis

smFRET experiments were performed using a custom-built objective-type total-internal-reflection fluorescence (TIRF) microscope (*16*). Light from a green laser (532 nm; Samba; Cobolt) and a red laser (635 nm; CUBE 635-30E, Coherent) was combined using a dichroic mirror coupled into a fiber-optic cable focused onto the rear focal plane of a 100x oil-immersion objective (numerical aperture 1.4; Olympus) and was displaced off the optical axis, such that the incident angle at the oil-glass interface of a stage-mounted observation chamber exceeded the critical angle, thereby creating an

exponentially decaying evanescent wave (*17*). Alternating-laser excitation (ALEX; *12*) was implemented by directly modulating the green and red lasers using an acousto-optical modulator (1205C, Isomet).

Fluorescence emission was collected from the objective, was separated from excitation light using a dichroic mirror (545 nm/650 nm, Semrock) and emission filters (545 nm LP, Chroma; and 633/25 nm notch filter, Semrock), was focused on a slit to crop the image, and then was spectrally separated (using a dichroic mirror; 630 nm DLRP, Omega) into donor and emission channels focused side-by-side onto an electron-multiplying charge-coupled device camera (EMCCD; iXon 897; Andor Technology). A motorized x/y-scanning stage with continuous reflective-interface feedback focus (MS-2000; ASI) was used to control the sample position relative to the objective.

All data acquisition was carried out at 22°C. For all TIRF-ALEX experiments except those in Fig. 3D, laser powers were 4 mW (532 nm laser) and 0.75 mW (635 nm laser), and data were collected for 20 s using a frame rate of 1 frame per 20 ms. For experiments in Fig. 3D, laser powers were 1 mW (532 nm laser) and 0.3 mW (635 nm laser), and data were collected for 50 s using a frame rate of 1 frame per 100 ms.
Fluorescence-emission intensities in donor-emission (green) and acceptor-emission (red) channels were detected using the peak-finding algorithm of the MATLAB (MathWorks) software package Twotone-ALEX, as described (*16*). Peaks detected in both emission channels (i.e., peaks for molecules containing both donor and acceptor probes) were fitted with two-dimensional Gaussian functions to extract background-corrected intensity-vs.-time trajectories for donor-emission intensity upon donor excitation (I_{DD}), acceptor-emission intensity upon donor excitation (I_{DA}), and acceptor-emission intensity upon acceptor excitation (I_{AA}), as described (*16*). Intensity-vs.-time trajectories were curated to exclude trajectories exhibiting I_{DD} <100 or >1,000 counts or I_{AA} <200 or >1,000 counts, trajectories exhibiting multiple-step donor or acceptor photobleaching, trajectories exhibiting donor or acceptor photobleaching in frames 1-20, trajectories exhibiting donor or acceptor photoblinking, trajectories exhibiting E* values < 0.3 (inferred to be donor-only complexes or improperly assembled complexes), and portions of trajectories following donor or acceptor photobleaching.

Intensity-vs.-time trajectories were used to calculate trajectories of apparent donor-acceptor smFRET efficiency (E*) and donor-acceptor stoichiometry (S), as described (*12, 15*):

$E^* = I_{DA}/ (I_{DD} + I_{DA})$
$S = (I_{DA} + I_{DD})/ (I_{DD} + I_{DA} + I_{AA})$

E*-vs.-S plots were prepared, S values were used to distinguish species containing only donor, only acceptor, and both donor and acceptor, and E* histograms were prepared for species containing both donor and acceptor, as described (*12, 15*), and fitted to Gaussian distributions in Origin (Origin Lab). The resulting histograms provide equilibrium population distributions of E* states and, for each E* state, define mean E* (Figs. 1C, 3A, S5, S6 and S9B; gray bars and inset).

E*-vs-time trajectories that, on visual inspection, exhibited transitions between distinct E* states and exhibited anti-correlated changes in DD (donor excitation-donor emission) and DA (donor excitation-acceptor emission) channels were identified. For experiments in Figs. 1C, S5 and S9, few traces (<3%) showed dynamic behavior. For experiments in Figs. 2, S6 and S9, ~10% to ~60% of traces showed dynamic behavior. Dynamic E*-vs-time trajectories were analyzed globally to identify E* states by use of Hidden Markov Modelling (HMM) as implemented the Matlab (MathWorks) software package ebFRET (*18*), essentially as described (*3, 18*). E*-vs-time trajectories were fitted to a two-state HMM model, E*-values from the fitted model were extracted, were plotted using Origin (Origin Lab), and were fitted to Gaussian distributions using Origin (Figs. 2B, 3B and S6; colored curves). The resulting histograms provide equilibrium population distributions of E* states and, for each E* state, define mean E* (Figs. 2B, 3B and S6; colored bars and inset).

E* values were corrected, and accurate donor-acceptor efficiencies (E_a) and donor-acceptor distances (R) were calculated (Table S1) as described previously (*4*). From the accurate FRET efficiencies (E_a), distances (R; Table S1) were estimated using:

$$R = R_0 [(1/E)-1]^{1/6}$$

where R_0 is the Főrster parameter for the donor/acceptor FRET pair [52 Å for DL550-DL650 FRET pair (*19*); 50 Å for DL550-Alexa647 FRET pair; calculated as: $R_0 = 9780(n^{-4}\kappa^2 Q_D J)^{1/6}$ Å, where n is the refractive index of the medium, κ^2 is the orientation factor relating donor and acceptor transition dipoles (approximated as 2/3, noting that all mean E values are <0.5; *20*), Q_D is the donor quantum yield, and J is the overlap integral of donor emission and acceptor excitation].

Dwell times for E* states were extracted from HMM fits to E*-vs-time trajectories and were binned and plotted as distribution histograms in Origin (Fig. S5). For experiments in Figs. 2, 3B and S6, the rate of TL closing (k_{close}) and the rate of TL opening (k_{open}) were estimated from single-exponential fits to the open-TL and closed-TL dwell-time-distribution histograms, respectively (Figs. 2D, 3C, and S6).

For experiments in Figs. 4, S9, and S10, the open-TL dwell-time-distribution histograms were fit to a single-exponential function, and the closed dwell-time-distribution histograms were fit to a bi-exponential function with most events corresponding to short dwells (~60 ms; ~90%) and some events corresponding to longer dwells (~400 ms; ~10%). The rate of TL opening (k_{open}) and the rate of TL closing (k_{close}) were estimated from exponential fits to closed and open dwell time distribution histograms, respectively, as described above (Fig. S9D and S10).

The on-rate for ATP binding, the off-rate for ATP unbinding and the equilibrium dissociation constant for ATP binding were estimated from the TL-closing and TL-opening rates (assuming TL closing events occur upon NTP-binding events, and TL-opening events occur upon NTP-unbinding events), as follows (Fig. 2E, 3C, S9D):

$$k_{close} = k_{on} * [ATP]$$
$$k_{open} = k_{off}$$
$$K_d = k_{off}/k_{on}$$

For experiments in Figs. 4 and S10, ~25% of molecules exhibited transitions between distinct E* states and exhibited anti-correlated changes in the DD (donor excitation-donor emission) and DA (donor excitation-acceptor emission) channels, upon addition of 5 μM ATP. One TL-closing step (transition from open-TL state to closed-TL state) through one TL-opening step (transition from closed-TL to open-TL state) was defined to constitute a TL closing-opening event. For each experiment in Fig. 4 (template directing addition of 1A, 2A, 3A, or 4A), a manual counting of numbers of TL closing-opening events was performed, and numbers of TL closing-opening events were plotted as a histogram in Origin. Rare complexes (<1%) that showed TL closing-opening events prior to ATP addition were excluded from the analysis. TL-closing and TL-opening rates were estimated as described above (Fig. S9). Molecules which did not show any transitions may include time traces where all transitions are missed or molecules which were unresponsive to addition of 5 μM ATP.

Supplementary References:

1. J.W. Chin, S.W. Santoro, A.B. Martin, D.S. King, L. Wang, P.G. Schultz, Addition of p-azido-L-phenylalanine to the genetic code of Escherichia coli. *J Am Chem Soc* **124**, 9026-9027 (2002).
2. A. Chakraborty, D. Wang, Y.W. Ebright, Y. Korlann, E. Kortkhonjia, T. Kim, S. Chowdhury, S. Wigneshweraraj, H. Irschik, R. Jansen, B.T. Nixon, J. Knight, S. Weiss, R.H. Ebright, Opening and closing of the bacterial RNA polymerase clamp. *Science* **337**, 591-595 (2012).
3. D. Duchi, A. Mazumder, A.M. Malinen, R.H. Ebright, A.N. Kapanidis, The RNA polymerase clamp interconverts dynamically among three states and is stabilized in a partly closed state by ppGpp. *Nucleic Acids Res* **46**, 7284-7295 (2018).
4. W. Lin, K. Das, D. Degen, A. Mazumder, D. Duchi, D. Wang, Y.W. Ebright, R.Y. Ebright, E. Sineva, M. Gigliotti, A. Srivastava, S. Mandal, Y. Jiang, Y. Liu, R. Yin, Z. Zhang, E.T. Eng, D. Thomas, S. Donadio, H. Zhang, C. Zhang, A.N. Kapanidis, R.H. Ebright, Structural basis of transcription inhibition by Fidaxomicin (Lipiarmycin A3). *Mol Cell* **70**, 60-71 e15 (2018).
5. B.P. Hudson, J. Quispe, S. Lara-Gonzalez, Y. Kim, H.M. Berman, E. Arnold, R.H. Ebright, C.L. Lawson, Three-dimensional EM structure of an intact activator-dependent transcription initiation complex. *Proc Natl Acad Sci U S A* **106**, 19830-19835 (2009).
6. C.E. Vrentas, T. Gaal, W. Ross, R.H. Ebright, R.L. Gourse, Response of RNA polymerase to ppGpp: requirement for the omega subunit and relief of this requirement by DksA. *Genes Dev* **19**, 2378-2387 (2005).
7. J. Sambrook, D. Russell, Molecular cloning: A laboratory manual (Cold Spring harbour, NY, Cold Spring Haror Laboratory) (2001).
8. W. Niu, Y. Kim, G. Tau, T. Heyduk, R.H. Ebright, Transcription activation at class II CAP-dependent promoters: two interactions between CAP and RNA polymerase. *Cell* **87**, 1123-1134 (1996).
9. D. Degen, Y. Feng, Y. Zhang, K.Y. Ebright, Y.W. Ebright, M. Gigliotti, H. Vahedian-Movahed, S. Mandal, M. Talaue, N. Connell, E. Arnold, W. Fenical, R.H. Ebright, Transcription inhibition by the depsipeptide antibiotic salinamide A. *eLife* **3**, e02451 (2014).
10. Y. Zhang, D. Degen, M.X. Ho, E. Sineva, K.Y. Ebright, Y.W. Ebright, V. Mekler, H. Vahedian-Movahed, Y. Feng, R. Yin, S. Tuske, H. Irschik, R. Jansen, S. Maffioli, S. Donadio, E. Arnold, R.H. Ebright, GE23077 binds to the RNA polymerase 'i' and 'i+1' sites and prevents the binding of initiating nucleotides. *eLife* **3**, e02450 (2014).
11. J. Mukhopadhyay, V. Mekler, E. Kortokhonjia, A.N. Kapanidis, Y.W. Ebright, R.H. Ebright, Fluorescence resonance energy transfer (FRET) in analysis of transcription-complex structure and function. *Methods Enzymol* **371**,144-159 (2003).
12. A.N. Kapanidis, N.K. Lee, T.A. Laurence, S. Doose, E. Margeat, S. Weiss, Fluorescence-aided molecule sorting: analysis of structure and interactions by alternating-laser excitation of single molecules. *Proc Natl Acad Sci U S A* **101**, 8936-8941 (2004).
13. J. Mukhopadhyay, E. Sineva, J. Knight, R.M. Levy, R.H. Ebright, Antibacterial peptide microcin J25 inhibits transcription by binding within and obstructing the RNA polymerase secondary channel. *Mol Cell* **14**, 739-751 (2004).
14. Ebright, R., Ebright, Y., Mandal, S., Wilde, R., and Li, Shengjian. (2018) Antibacterial agents: N(alpha)-aroyl-N-aryl-phenylalaninamides. *US Patent* US9919998.36.
15. N.K. Lee, A.N. Kapanidis, Y. Wang, X. Michalet, J. Mukhopadhyay, R.H. Ebright, S. Weiss, Accurate FRET measurements within single diffusing biomolecules using alternating-laser excitation. *Biophys J* **88**, 2939-2953 (2005).

16. J. Holden, S. Uphoff, J. Hohlbein, D. Yadin, L. Le Reste, O.J. Britton, A.N. Kapanidis, Defining the limits of single-molecule FRET resolution in TIRF microscopy. *Biophys J* **99**, 3102-3111 (2010).

17. D. Axelrod, N.L. Thompson, T.P. Burghardt, Total internal inflection fluorescent microscopy. *J Microsc* **129**, 19-28 (1983).

18. J.W. van de Meent, J.E. Bronson, C.H. Wiggins, R.L. Gonzalez, Jr., Empirical Bayes methods enable advanced population-level analyses of single-molecule FRET experiments. *Biophys J* **106**, 1327-1337 (2014).

19. S. Schulz, A. Gietl, K. Smollett, P. Tinnefeld, F. Werner, D. Grohmann, TFE and Spt4/5 open and close the RNA polymerase clamp during the transcription cycle. *Proc Natl Acad Sci U S A* **113**, E1816-1825 (2016).

20. P. Wu, L. Brand, Orientation factor in steady-state and time-resolved resonance energy transfer measurements. *Biochemistry* **31**, 7939-7947 (1992).

21. S. Tuske, S. G. Sarafianos, X. Wang, B. Hudson, E. Sineva, J. Mukhopadhyay, J. J. Birktoft, O. Leroy, S. Ismail, A. D. Clark, Jr., C. Dharia, A. Napoli, O. Laptenko, J. Lee, S. Borukhov, R. H. Ebright, E. Arnold, Inhibition of bacterial RNA polymerase by streptolydigin: stabilization of a straight-bridge-helix active-center conformation. *Cell* **122**, 541-552 (2005).

22. D. G. Vassylyev, M. N. Vassylyeva, J. Zhang, M. Palangat, I. Artsimovitch, R. Landick, Structural basis for substrate loading in bacterial RNA polymerase. *Nature* **448**, 163-168 (2007).

23. Y. Zuo, T.A. Steitz, Crystal structures of the E. coli transcription initiation complexes with a complete bubble. *Mol Cell* **58**, 534-540 (2015).

24. Y. Yang, V.C. Darbari, N. Zhang, D. Lu, R. Glyde, Y.P. Wang, J.T. Winkelman, R.L. Gourse, K.S Murakami, M. Buck, X. Zhang, Transcription. Structures of the RNA polymerase-sigma54 reveal new and conserved regulatory strategies. *Science* **349**, 882-885 (2015).

Supplementary Figures

Fig. S1. Use of smFRET to detect and characterize TL closing and opening in solution
(A) Open-TL (left subpanel) and closed-TL (right subpanel) conformational states as observed in crystal structures of *E. coli* RNAP (*23,24*; PDB 5BYH and PDB 4YLN). Pink ribbon, species-specific sequence insertion 3 (SI3) in open-TL state. Light green ribbon, SI3 in closed-TL state. , pink. Other colors as in Fig. 1A.
(B) Measurement of smFRET between first fluorescent probe incorporated at β' residue 942 in tip of *E. coli* RNAP TL (red sphere for open-TL state; green sphere for closed-TL) and second fluorescent probe incorporated at template-strand position +12 of downstream DNA (pink sphere). Inter-residue distances are ~41 Å for open-TL state and ~46 Å for closed-TL state. Other colors as in A.

Fig. S2. Nucleic-acid scaffolds
(A) Nucleic-acid scaffolds for reconstitution of post-translocated TEC with 9 bp DNA-RNA hybrid with non-extendable RNA 3' end (3'-deoxy). Black, DNA template strand; gray, DNA nontemplate strand; blue, RNA; cyan, non-extendable RNA 3' end; orange, fluorescent-probe-labelled nucleotide.
(B) Nucleic-acid scaffold for reconstitution of pre-translocated TEC with 10 bp DNA-RNA hybrid. Black, DNA template strand; gray, DNA nontemplate strand; blue, RNA; orange, fluorescent-probe-labelled nucleotide.
(C) Nucleic-acid scaffolds for reconstitution of post-translocated TEC with 9 bp DNA-RNA hybrid and extendable RNA 3' end (3'-hydroxy). Black, DNA template strand; gray, DNA nontemplate strand; blue, RNA.

Fig. S3. Incorporation of fluorescent probe into RNAP: singly-labelled RNAP derivative
(A) Products of labelling reaction of RNAP derivative containing 4-azidophenylalanine (AzF) at position 942 of β’ subunit with Dylight 550 phosphine, as detected by Coomassie staining (left) and fluorescent scanning in donor-emission channel (532 nm excitation and 580 nm emission bandpass filters; right). Fluorescent labelling is observed only for the β’ subunit.
(B) Labelling efficiencies and labelling specificities (see Materials and Methods).
(C) Transcriptional activities of fluorescent-probe-labelled RNAP.

Fig. S4. Use of smFRET to detect and characterize TL closing and opening in solution: confocal smFRET data
(A) smFRET data for TEC in post-translocated state with a donor fluorescent probe in RNAP TL and acceptor fluorescent probe at position +10 of DNA template strand DNA of nucleic-acid scaffold. Data are shown for absence of NTP (top) and presence of saturating concentration of complementary NTP (1 mM ATP; bottom). Histogram and Gaussian fit of E* provide mean E* values for open-TL (red line) and closed-TL (green line) states.
(B) As A, but for TEC in post-translocated state with donor fluorescent probe in RNAP TL and acceptor fluorescent probe at position +12 of DNA template strand of nucleic-acid scaffold.
Fig. S3. Use of smFRET to detect and characterize TL closing and opening in solution: TL conformation in TEC in pre-translocated state

Fig. S5. smFRET data for TEC in pre-translocated state in absence of NTP
Left subpanel, representative time traces of donor-acceptor FRET efficiency, E*, showing open-TL state (red). Right subpanel, histogram and Gaussian fit of E*, showing mean E* value of open-TL state (red line). R, mean donor-acceptor distance.

Fig. S6. TL closing and opening occur on millisecond timescales: additional smFRET data in presence of sub-saturating concentrations of complementary NTP
(A) smFRET data for TEC in post-translocated state in presence of each of four sub-saturating concentrations of complementary NTP (10, 20, 40, and 80 μM ATP). Left top, representative time trace of donor emission (purple) and acceptor emission (orange). Left, bottom, representative time trace of donor-acceptor FRET efficiency, E*, showing hidden-Markov-model (HMM)-assigned open-

TL states (red), closed-TL states (green), and interstate transitions (blue). Right, histograms and Gaussian fits of E* show mean E* values of open-TL (red lines) and closed-TL (green lines) states. P, subpopulation percentage; R, mean donor-acceptor distance.
(B) Dwell-time distributions of open-TL states.
(C) Dwell-time distribution of closed-TL states.

Fig. S7. TL closing and opening can provide a checkpoint for NTP complementarity: additional smFRET data on effects of NTP complementarity on TL conformation
(A) Effects of complementary and non-complementary NTPs on TL conformation with template directing binding of ATP. Histograms and Gaussian fits of E* show mean E* values of open-TL (red line) and closed-TL (green line) states.
(B) As A, but with template directing binding of GTP.
(C) As A, but with template directing binding of CTP.
(D) As A, but with template directing binding of UTP.

Fig. S8. Incorporation of fluorescent probes into RNAP: doubly-labelled RNAP derivative
(A) Products of stochastic labelling reaction of RNAP derivative containing 4-azidophenylalanine (AzF) at position 942 of β' subunit and position 267 of β subunit with Dylight 550 phosphine and Dylight 650 phosphine, as detected by Coomassie staining (left), fluorescent scanning in donor-emission channel (D; 532 nm excitation and 580 nm emission bandpass filters; center) and fluorescent scanning in acceptor-emission channel (A; 633 nm excitation and 670 nm emission bandpass filters; right). Fluorescent labelling is observed only for β' and β subunits.
(B) Labelling efficiencies and labelling specificities (see Materials and Methods).
(C) Transcriptional activities of fluorescent-probe-labelled RNAP.

Fig. S9. Use of smFRET to detect and characterize TL closing and opening in solution: doubly-labelled RNAP derivative
(A) Measurement of smFRET between first fluorescent probe incorporated β' residue 942 at tip of RNAP TL (red sphere for open-TL state; green sphere for closed-TL) and second fluorescent probe incorporated RNAP β subunit residue 567 (pink sphere). Inter-residue distances are ~31 Å for open-TL state and ~52 Å for closed-TL state. Open-TL (red) and closed-TL (green) conformational states are as observed in crystal structures (*21,22;* PDB 1ZYR and PDB 2O5J).. Gray and red ribbon, RNAP trigger helices and TL in open-TL state; gray and green ribbon, RNAP trigger helices and TL in closed-TL state; gray and black sticks, DNA non-template and template strands; blue sticks, RNA 3' nucleotide; cyan sticks, incoming NTP; purple spheres, catalytic Mg^{2+} ions Mg^{2+}(I) and Mg^{2+}(II).
(B) smFRET data for TEC in post-translocated state in absence of NTP (top) and in presence of saturating concentration of complementary NTP (1 mM ATP; bottom). Histograms and Gaussian fits of E* show mean E* values for open-TL (red line) and closed-TL (green line) states. R, mean donor-acceptor distance.
(C) smFRET data for TEC in post-translocated state in presence of each of four sub-saturating concentrations of complementary NTP (10, 20, 40, and 80 μM ATP). Representative time traces of donor-acceptor FRET efficiency, E*, showing hidden-Markov-model (HMM)-assigned open-TL states (red), closed-TL states (green), and interstate transitions (blue).
(D) TL-closing rate (k_{close}), TL-opening rate (k_{open}), ATP on-rate (k_{on}), and ATP off-rate (k_{off}) from experiments of A-C.

Fig. S10. One TL closing-opening cycle typically occurs in each nucleotide addition in transcription elongation: TL-closing and TL-opening dynamics during nucleotide addition
(A) Dwell-time distributions for open-TL (left) and closed-TL (right) states during nucleotide addition.
(B) Number of observed TL closing-opening events for templates directing 1, 2, 3, or 4 additions of A (i.e., n = 1, n =2, , n =3, and n = 4).

Table S1: Apparent FRET (E*), distances (R) and subpopulation percentages (P) for elongation complexes: DL 550 on tip of trigger loop (β' residue 942) and Alexa 647 on +12 position of the template strand

elongation complex	substrate (μM)	figure	E* mean (±SEM)	R mean (±SEM) (Å)	P (subpopulation) (%)
pre-translocated TEC	ATP (0 μM)	S2	0.62(±0.001)	48.5(±0.06)	open (100%)
post-translocated TEC	ATP (0 μM)	1F	0.61(±0.001)	48.8(±0.05)	open (100%)
post-translocated TEC	ATP (10 μM)	S3	0.60(±0.000)<br>0.46(±0.000)	49.2(±0.02)<br>55.0(±0.01)	open (83±0.5%)<br>closed (17±0.1%)
post-translocated TEC	ATP (20 μM)	2A	0.60(±0.000)<br>0.46(± 0.000)	49.2(±0.02)<br>55.0(±0.01)	open (68±0.3%)<br>closed (32±0.2%)
post-translocated TEC	ATP (40 μM)	S3	0.61(±0.000)<br>0.47(±0.000)	48.8(±0.02)<br>54.5(±0.03)	open (60±0.7%)<br>closed (40±0.3%)
post-translocated TEC	ATP (80 μM)	S3	0.60(±0.000)<br>0.46(±0.000)	49.2(±0.01)<br>55.0(±0.03)	open (40±0.3%)<br>closed (60±0.3%)
post-translocated TEC	ATP (1000 μM)	1F	0.51(±0.000)		
post-translocated TEC	GTP (1000 μM)	3A	0.62(±0.000)	48.5(±0.03)	open (100%)
post-translocated TEC	UTP (1000 μM)	3A	0.60(±0.001)	49.2(±0.04)	open (100%)
post-translocated TEC	CTP (1000 μM)	3A	0.60(± 0.000)	49.2(±0.03)	open (100%)
post-translocated TEC	ADP (1000 μM)	3B	0.60(±0.001)<br>0.51(±0.000)	49.2(±0.04)<br>52.8(±0.03)	open (80±0.8%)<br>closed (20±0.3%)
post-translocated TEC	AMP (1000 μM)	3B	0.60(±0.001)	48.5(±0.03)	open (100%)
post-translocated TEC	2'-dATP (1000 μM)	3C	0.61(±0.000)<br>0.45(±0.001)	48.8(±0.03)<br>55.5(±0.07)	open (85±0.5 %)<br>closed (15±0.2%)

Table S2: Apparent FRET (E*), distances (R) and subpopulation percentages (P) for elongation complexes: DL550 / DL650 on tip of trigger loop (β' residue 942) and β subunit (residue 267)

elongation complex	substrate (μM)	figure	E* mean (±SEM)	R mean (±SEM) (Å)	P (subpopulation) (%)
post-translocated TEC	ATP (0 μM)	S9	0.59(±0.000)	57.9(±0.01)	open (100%)
post-translocated TEC	ATP (1000 μM)	S9	0.48(±0.000)	53.1(±0.01)	closed (100%)

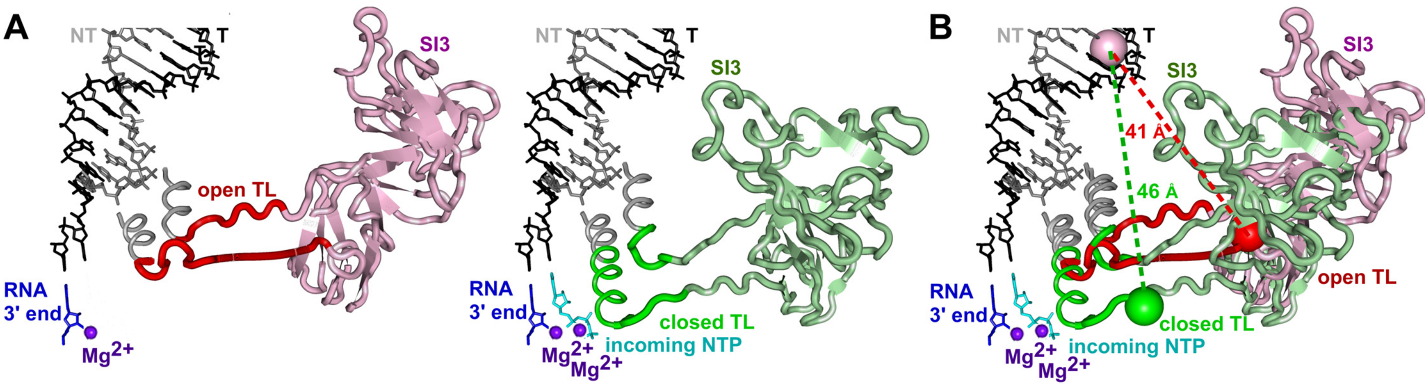
A
NT
T
SI3
open TL
RNA
3' end
Mg2+
NT
T
SI3
closed TL
RNA
3' end
Mg2+
Mg2+
incoming NTP
B
NT
T
SI3
SI3
41 Å
46 Å
open TL
closed TL
RNA
3' end
Mg2+
Mg2+
incoming NTP

Fig. S1

A **nucleic acid sequences for post-translocated TEC (9 bp DNA/RNA hybrid) with non-extendable RNA**

template directs ATP binding

5'-ACGCCAGACAGGACCTCAGTCCG-3'
3'-GCCGCGCGCTTGCGGTCTGTCCTGGAGTCAGGC-5'
5'-GAGUCUGCGGCGCGCG-3'

Alexa 647 on +10 of template strand
template directs ATP binding

5'-ACGCCAGACAGGACCTCAGTCCG-3'
3'-GCCGCGCGCTTGCGGTCTGTCCTGGAGTCAGGC-5'
5'-GAGUCUGCGGCGCGCG-3'

Alexa 647 on +12 of template strand
template directs ATP binding

5'-ACGCCAGACAGGACCTCAGTCCG-3'
3'-GCCGCGCGCTTGCGGTCTGTCCTGGAGTCAGGC-5'
5'-GAGUCUGCGGCGCGCG-3'

Alexa 647 on +12 of template strand
template directs UTP binding

5'-ACGCCAGACAGGACCTCAGTCCG-3'
3'-GCCGCGCGCATGCGGTCTGTCCTGGAGTCAGGC-5'
5'-GAGUCUGCGGCGCGCG-3'

Alexa 647 on +12 of template strand
template directs GTP binding

5'-ACGCCAGACAGGACCTCAGTCCG-3'
3'-GCCGCGCGCCTGCGGTCTGTCCTGGAGTCAGGC-5'
5'-GAGUCUGCGGCGCGCG-3'

Alexa 647 on +12 of template strand
template directs CTP binding

5'-ACGCCAGACAGGACCTCAGTCCG-3'
3'-GCCGCGCGCGTGCGGTCTGTCCTGGAGTCAGGC-5'
5'-GAGUCUGCGGCGCGCG-3'

B **nucleic acid sequence for pre-translocated TEC (10 bp DNA/RNA hybrid) with extendable RNA**

Alexa 647 on +12 of template strand

5'-ACGCCAGACAGGACCTCAGTCCG-3'
3'-GCCGCGCGCTTGCGGTCTGTCCTGGAGTCAGGC-5'
5'-GAGUCUGCGGCGCGCGA-3'

C **nucleic acid scaffold sequences for post-translocated TEC (9 bp DNA/RNA hybrid) with extendable RNA**

template directs 1 nucleotide (A) addition

5'-CGCCAGACAGGACCTCAGTCCG-3'
3'-GCCGCGCGCTGCGGTCTGTCCTGGAGTCAGGC-5'
5'-GAGUCUGCGGCGCGCG-3'

template directs 2 nucleotide (A) addition

5'-ACGCCAGACAGGACCTCAGTCCG-3'
3'-GCCGCGCGCTTGCGGTCTGTCCTGGAGTCAGGC-5'
5'-GAGUCUGCGGCGCGCG-3'

template directs 3 nucleotide (A) addition

5'-AACGCCAGACAGGACCTCAGTCCG-3'
3'-GCCGCGCGCTTTGCGGTCTGTCCTGGAGTCAGGC-5'
5'-GAGUCUGCGGCGCGCG-3'

template directs 4 nucleotide (A) addition

5'-AAACGCCAGACAGGACCTCAGTCCG-3'
3'-GCCGCGCGCTTTTGCGGTCTGTCCTGGAGTCAGGC-5'
5'-GAGUCUGCGGCGCGCG-3'

Fig. S2

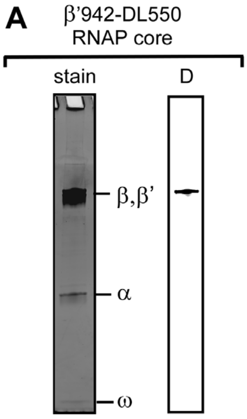

B

RNAP	labeling efficiency (%)	labeling specificity (%)
β'942-DL550 RNAP core	60	>95

C

RNAP	specific activity (%)
β'942-DL550 RNAP core	70

Fig. S3.

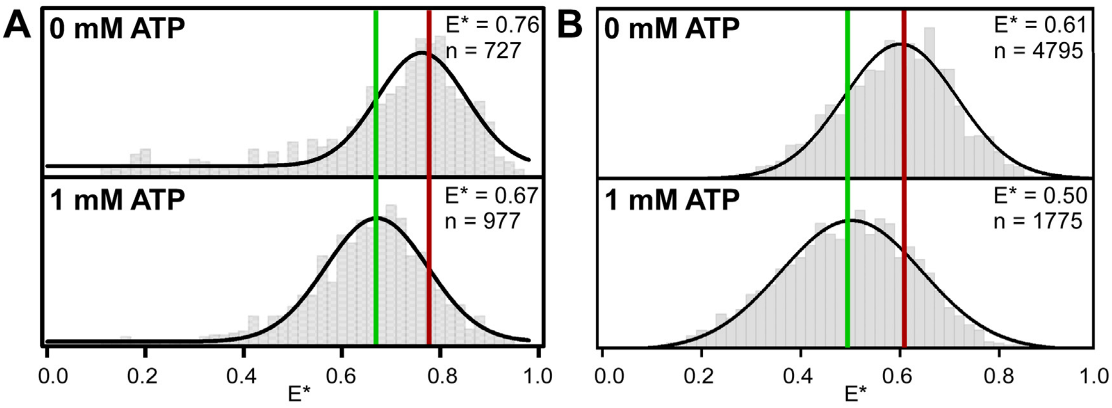
A
0 mM ATP
E* = 0.76
n = 727
1 mM ATP
E* = 0.67
n = 977
0.0
0.2
0.4
0.6
0.8
1.0
E*
B
0 mM ATP
E* = 0.61
n = 4795
1 mM ATP
E* = 0.50
n = 1775
0.0
0.2
0.4
0.6
0.8
1.0
E*

Fig. S4.

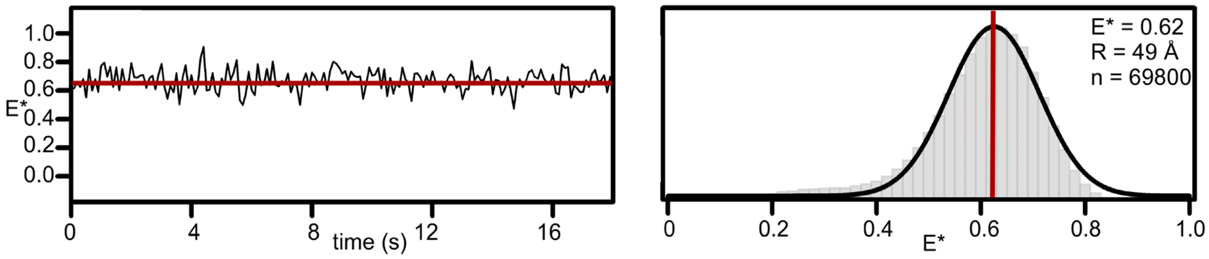

1.0
0.8
0.6
0.4
0.2
0.0
E*
0
4
8
12
16
time (s)
E* = 0.62
R = 49 Å
n = 69800
0
0.2
0.4
0.6
0.8
1.0
E*

Fig. S5.

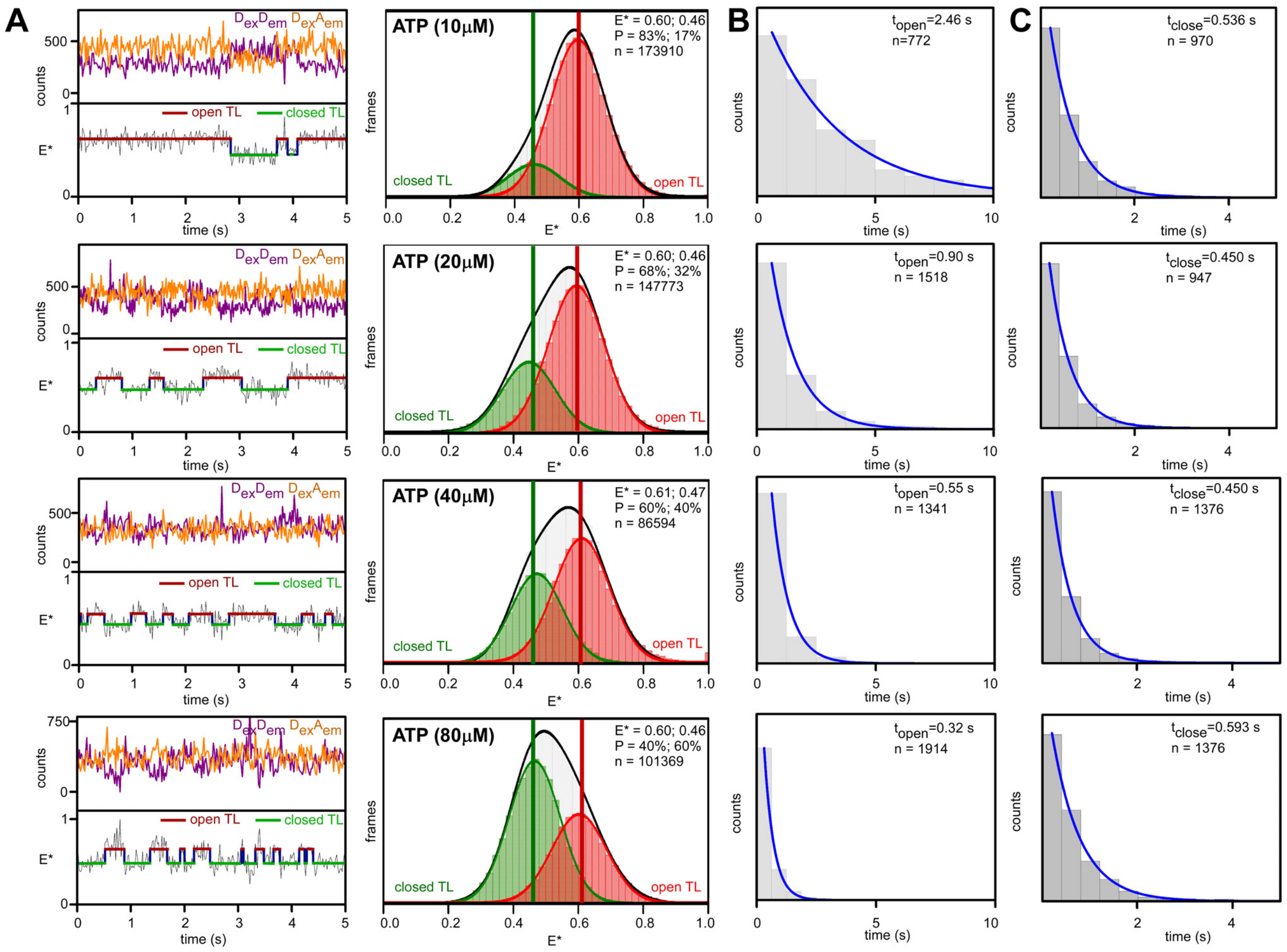
A
B
C
ATP (10μM)
E* = 0.60; 0.46
P = 83%; 17%
n = 173910
t_open=2.46 s
n=772
t_close=0.536 s
n = 970
ATP (20μM)
E* = 0.60; 0.46
P = 68%; 32%
n = 147773
t_open=0.90 s
n = 1518
t_close=0.450 s
n = 947
ATP (40μM)
E* = 0.61; 0.47
P = 60%; 40%
n = 86594
t_open=0.55 s
n = 1341
t_close=0.450 s
n = 1376
ATP (80μM)
E* = 0.60; 0.46
P = 40%; 60%
n = 101369
t_open=0.32 s
n = 1914
t_close=0.593 s
n = 1376
open TL
closed TL
counts
frames
E*
time (s)

Fig. S6.

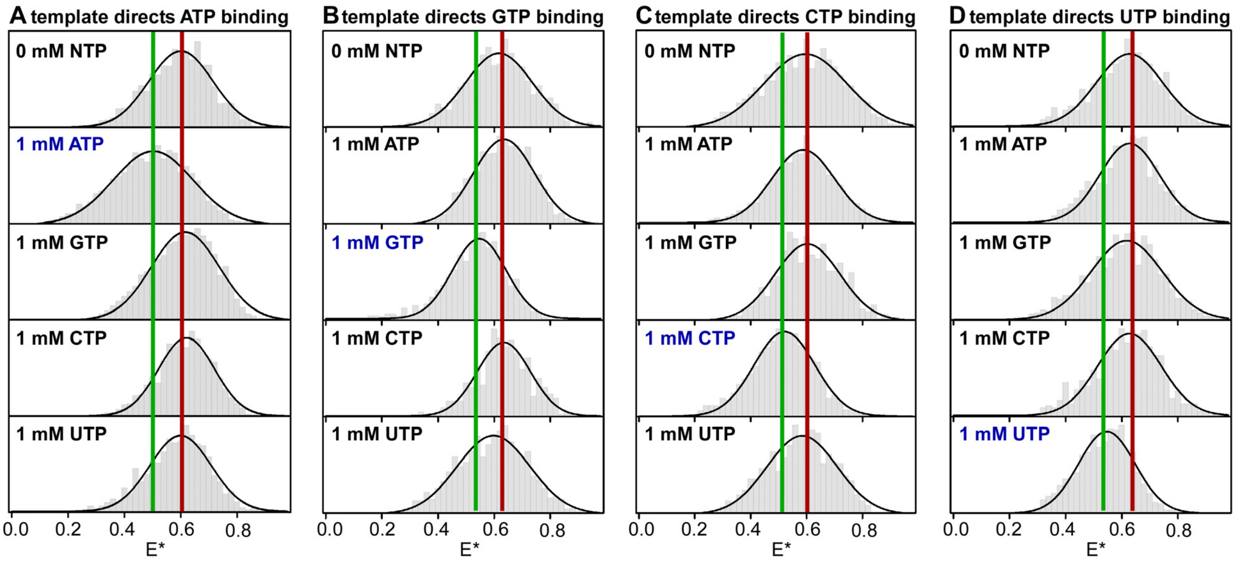

A template directs ATP binding
B template directs GTP binding
C template directs CTP binding
D template directs UTP binding
0 mM NTP
1 mM ATP
1 mM GTP
1 mM CTP
1 mM UTP
0.0
0.2
0.4
0.6
0.8
E*

Fig. S7.

A

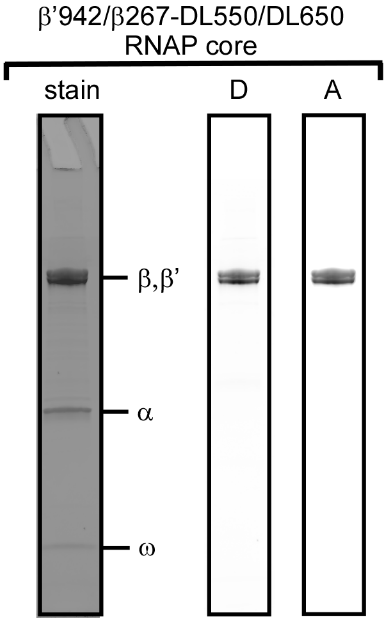

B

RNAP	labeling efficiency (%)	labeling specificity (%)
β'942/β267-DL550/DL650 RNAP core	50 (DL550); 80 (DL650)	>95

C

RNAP	specific activity (%)
β'942/β267-DL550/DL650 RNAP core	60

Fig. S8.

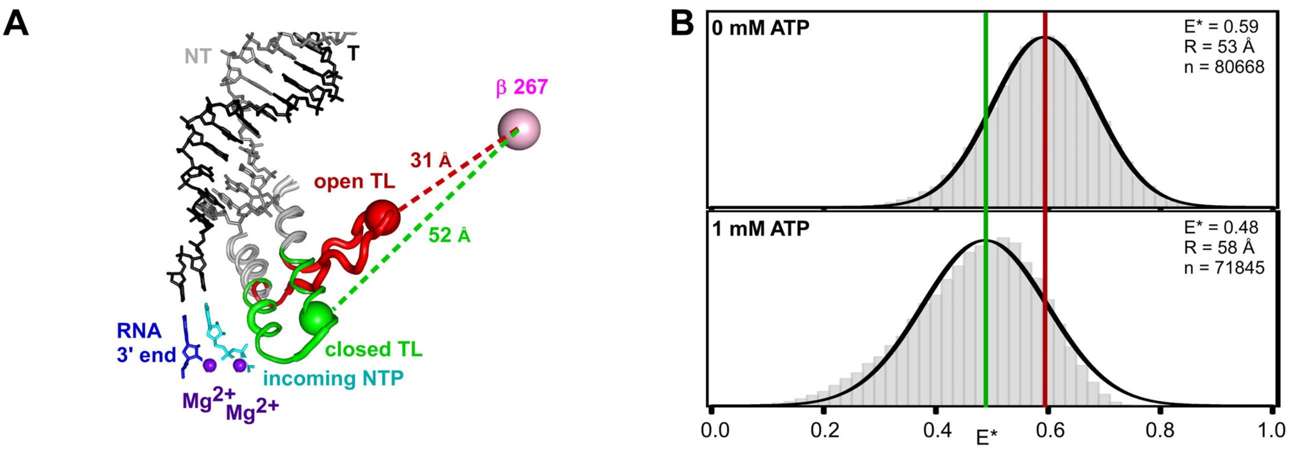

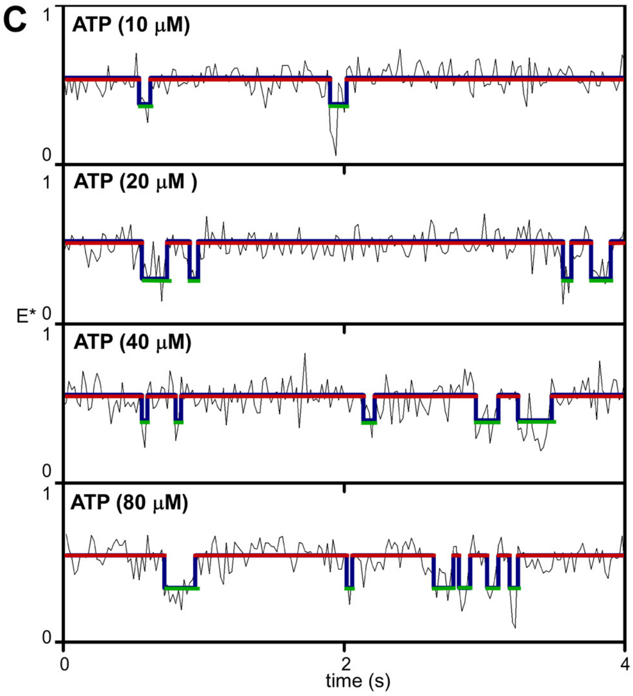

D

ATP (μM)	k_{close} (s^{-1})	k_{open} (s^{-1})	k_{on} ($\mu M^{-1}s^{-1}$)	k_{off} (s^{-1})
10	0.99 ± 0.05	18.8 ± 1.60 (90%)<br>2.80 ± 0.40 (10%)		
20	0.93 ± 0.06	21.7 ± 7.90 (67%)<br>2.70 ± 0.31 (33%)	0.038 ± 0.008	16.7 ± 0.60;<br>2.46 ± 0.12
40	1.57 ± 0.08	15.1 ± 0.90 (90%)<br>2.26 ± 0.36 (10%)		
80	2.40 ± 0.13	16.7 ± 1.40 (91%)<br>2.1 ± 0.24 (9%)		

Fig. S9.

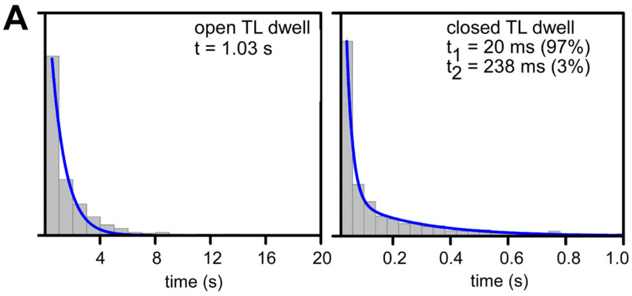

B

	events (1 to ≤n*)	events (>n; <10)	events (>10)
template directing 1 addition of A	23	30	6
template directing 2 additions of A	32	12	9
template directing 3 additions of A	31	8	2
template directing 4 additions of A	36	11	3
total	122	61	20

**n is the number of additions of A directed by the template (n = 1, n = 2, n =3, and n = 4 for templates directing 1, 2, 3, and 4 additions of A).*

Fig. S10.
